# Patients with low prognosis in ART: a Delphi consensus to identify potential clinical implications and measure the impact of POSEIDON criteria

**DOI:** 10.1186/s12958-024-01291-x

**Published:** 2024-10-10

**Authors:** Carlo Alviggi, Peter Humaidan, Robert Fischer, Alessandro Conforti, Michael H. Dahan, Antonio La Marca, Raoul Orvieto, Nikolaos P. Polyzos, Matheus Roque, Sesh K. Sunkara, Filippo Maria Ubaldi, Lan Vuong, Hakan Yarali, Thomas D’Hooghe, Salvatore Longobardi, Sandro C. Esteves

**Affiliations:** 1 Department of Public Health, University of Naples Federico II, Via Sergio Pansini, Naples, 80131 Italy; 2 The Fertility Clinic, Faculty of Health, Skive Regional Hospital, Aarhus University, Aarhus C, Denmark; 3 Fertility Centre Hamburg, Hamburg, Germany; 4 Department of Neuroscience, Reproductive Science and Odontomastology, University of Naples Federico II, Via Sergio Pansini, Naples, 80131 Italy; 5 Department of Obstetrics and Gynecology, McGill University, 888 De Maisonneuve Est., Montreal, QC H2L 4S8 Canada; 6 Department of Medical and Surgical Sciences for Mother, Child and Adult, University of Modena and Reggio Emilia, Modena, Italy; 7 Department of Obstetrics and Gynecology, Chaim Sheba Medical Center, Tel-Hashomer, Ramat Gan, 52621 Israel; 8 The Tarnesby-Tarnowski Chair for Family Planning and Fertility Regulation, Faculty of Medical and Health Science, Tel-Aviv University, Tel Aviv-Yafo, 6997801 Israel; 9 Dexeus Fertility, Dexeus University Hospital, Barcelona, 08028 Spain; 10 Faculty of Medicine and Health Sciences, University of Ghent, Gent, 9000 Belgium; 11 Department of Reproductive Medicine, Mater Prime, São Paulo, Brazil; 12 King’s Fertility, King’s College London, London, UK; 13 IVIRMA Global Research Alliance, GENERA, Clinica Valle Giulia, Rome, Italy; 14 Department of Obstetrics and Gynaecology, University of Medicine and Pharmacy at Ho Chi Minh City, Ho Chi Minh City, Vietnam; 15 Anatolia IVF and Women Health Centre, Ankara, Turkey; 16 Merck Healthcare KGaA, Darmstadt, Germany; 17 Department of Development and Regeneration, Laboratory of Endometrium, Endometriosis & Reproductive Medicine, KU Leuven, Leuven, Belgium; 18 Department of Obstetrics, Gynecology, and Reproductive Sciences, Yale University Medical School, New Haven, USA; 19 Global Clinical Development, Merck Serono S.p.A (an affiliate of Merck KGaA, Darmstadt 64293, Germany), Rome, 00176 Italy; 20 ANDROFERT, Andrology and Human Reproduction Clinic, Campinas, São Paulo, Brazil; 21 Faculty of Health, Aarhus University, Aarhus, Denmark

**Keywords:** Assisted reproductive technology (ART), Delphi consensus, Ovarian stimulation, POSEIDON Criteria, Low prognosis, Cumulative live birth rate (CLBR)

## Abstract

**Background:**

Currently, there is no consensus on the optimal management of women with low prognosis in ART. In this Delphi consensus, a panel of international experts provided real-world clinical perspectives on a series of literature-supported consensus statements regarding the overall relevance of the POSEIDON criteria for women with low prognosis in ART.

**Methods:**

Using a Delphi-consensus framework, twelve experts plus two Scientific Coordinators discussed and amended statements and supporting references proposed by the Scientific Coordinators (Round 1). Statements were distributed via an online survey to an extended panel of 53 experts, of whom 36 who voted anonymously on their level of agreement or disagreement with each statement using a six-point Likert-type scale (1 = Absolutely agree; 2 = More than agree; 3 = Agree; 4 = Disagree; 5 = More than disagree; 6 = Absolutely disagree) (Round 2). Consensus was reached if > 66% of participants agreed or disagreed.

**Results:**

The extended panel voted on seventeen statements and subcategorized them according to relevance. All but one statement reached consensus during the first round; the remaining statement reached consensus after rewording. Statements were categorized according to impact, low-prognosis validation, outcomes and patient management. The POSEIDON criteria are timely and clinically sound. The preferred success measure is cumulative live birth and key management strategies include the use of recombinant FSH preparations, supplementation with r-hLH, dose increases and oocyte/embryo accumulation through vitrification. Tools such as the ART Calculator and Follicle-to-Oocyte Index may be considered. Validation data from large, prospective studies in each POSEIDON group are now needed to corroborate existing retrospective data.

**Conclusions:**

This Delphi consensus provides an overview of expert opinion on the clinical implications of the POSEIDON criteria for women with low prognosis to ovarian stimulation.

**Supplementary Information:**

The online version contains supplementary material available at 10.1186/s12958-024-01291-x.

## Introduction

The management of patients with low prognosis in assisted reproductive technology (ART) represents a challenging topic for clinicians. Currently, there is no consensus regarding the optimal treatment for low prognosis patients in order to optimize ovarian response and increase delivery rate.

Women with low prognosis to ART are usually identified as poor ovarian responders (PORs). The European Society of Reproduction and Embryology (ESHRE) Bologna criteria defines a woman as a POR if at least two of the following features are present: advanced maternal age or other risk factor for POR; a previous cycle with POR; an abnormal ovarian reserve test (antral follicle count [AFC] < 5–7 follicles or anti-müllerian hormone [AMH] 0.05–1.1 ng/mL) [1]. Importantly, despite strong heterogeneity among the population included, these criteria are still able to identify women who will exhibit a poor response to exogenous gonadotropins [2].

Although the number of oocytes retrieved represents a crucial quantitative parameter for the prediction of in-vitro fertilization (IVF) success, other important qualitative parameters, such as age-related reduction in oocyte/embryo quality, embryo/blastocyst aneuploidy rate and age-related decrease in ovarian sensitivity to gonadotropins (hypo-responders) [3–7] should also be considered. With this in mind, the Patient-Oriented Strategies Encompassing IndividualizeD Oocyte Number (POSEIDON) criteria shifted the focus from POR to one of low prognosis, incorporating both quantitative and qualitative predictors of IVF success [2]. As a result, the POSEIDON criteria categorized low prognosis patients into four different groups according to age, ovarian reserve markers (AMH, AFC or both) and the number of oocytes retrieved in previous cycles of ovarian stimulation (Table 1), with each group representing a specific segment of prognosis defined in terms of cumulative live birth rate (CLBR) per started/aspirated cycle. The POSEIDON criteria may serve as a guide for personalizing treatment and introduces a new endpoint for success (the ability to retrieve the number of oocytes necessary to obtain at least one euploid embryo) and provide a more nuanced picture of POR that can be used to guide the management of women with low prognosis [4, 8]. Notably, the POSEIDON criteria do not recommend that pre-implantation genetic testing for aneuploidy be routinely performed on every patient and the criteria can be used in conjunction with the ART Calculator, which identified female age and type of sperm used for IVF/ICSI as the relevant predictors concerning blastocyst euploidy from approximately 350 infertile couples undergoing IVF/ICSI and PGT-A [9–11].

**Table 1:** The POSEIDON Criteria

	POSEIDON Group 1a	POSEIDON Group 1b	POSEIDON Group 2a	POSEIDON Group 2b	POSEIDON Group 3	POSEIDON Group 4
Age	< 35 years	< 35 years	≥ 35 years	≥ 35 years	< 35 years	≥ 35 years
Ovarian biomarkers						
AFC	≥ 5	≥ 5	≥ 5	≥ 5	< 5	< 5
AMH	≥ 1.2 ng/mL	≥ 1.2 ng/mL	≥ 1.2 ng/mL	≥ 1.2 ng/mL	< 1.2 ng/mL	< 1.2 ng/mL
Oocytes in a previous cycle	< 4	4–9	< 4	4–9	-	-

The POSEIDON criteria to identify and stratify infertility patients with “expected” or “unexpected” impaired ovarian response to exogenous gonadotropins undergoing ART. Four distinct groups of low prognosis patients can be established based on quantitative and qualitative parameters: the age of the patient and the expected embryo aneuploidy rate; ovarian biomarkers (AFC and/or AMH); and the ovarian response of the patient in terms of oocyte quantity from a previous cycle of stimulation (POSEIDON groups 1a, 1b, 2a and 2b only*). Groups 1 & 2 include patients with adequate ovarian reserve parameters and an unexpected POR after ovarian stimulation, whereas Groups 3 & 4 include patients with POR parameters who present with an expected POR after ovarian stimulation. Groups 1 & 3 are younger women (< 35 years) who have a low risk of aneuploidy, whereas Groups 2 & 4 are older (≥ 35 years) and have a high risk of aneuploidy. Low oocyte number leads to fewer embryos and reduced cumulative delivery rate per cycle compared with normal of high responders of a similar age category; therefore, maximizing oocyte yield to increase the likelihood of having at least one euploid embryo for transfer is needed for all four groups

AFC, antral follicle count. AMH, anti-Müllerian hormone

Table adapted from Esteves et al. Front. Endocrinol. 2018;9:461

In this study, a Delphi consensus was conducted to gather and evaluate expert opinion on the overall relevance of the POSEIDON criteria to identify potential clinical implications and measure its impact on the diagnosis of infertility in women with low prognosis.

## Methods

### Role of the sponsor

The Delphi consensus was coordinated by a healthcare consulting and training company (Sanitanova Srl, Milan, Italy). The consensus concept was initiated and funded by Merck KGaA, Darmstadt, Germany. The sponsor was involved early in the process, defining the overarching topic to be discussed, but did not participate in the development of the statements or in any of the meetings or discussions involved in developing the Delphi consensus. The statements were, therefore, developed independently of the industry sponsor. The authors from Merck KGaA, Darmstadt, Germany, were only involved in the development of the manuscript, critically revising it for important intellectual content, especially in the Introduction, Results and Discussion sections, but could not alter the consensus statements in any way.

### Consensus participants

The Delphi consensus involved a Scientific Board, comprising two Scientific Coordinators (**CA** and **SE**) and 12 additional experts selected on the basis of their publication records and relevant contributions at international medical congresses and meetings (Table 2). Each member of the Scientific Board suggested two or three additional experts (41 in total) who were invited to participate in the subsequent steps of the consensus process (Supplementary Table 1). The extended panel therefore comprised 53 experts: the 12 Scientific Board members (excluding the Scientific Coordinators) and 41 additional experts. The extended panel participating in the online survey comprised fertility experts from a number of different regions, including Europe, Asia, North America and South America.

**Table 2:** Participants involved in Rounds 1–3 of the consensus

Name	Country	Round 1 (WebEx meeting)*		Round 2 (Online survey)	Round 3 (WebEx meeting)
		29 March 2022 (PM)	1 April 2022 (PM)	May – June 2022 Statement 16 rewording and new vote September – October 2022	8 November 2022 (PM)
**Scientific Coordinators**					
Carlo Alviggi	Italy	X	X		X
Sandro Esteves	Brazil	X	X		X
**Scientific Board**					
Alessandro Conforti	Italy		X	X	X
Michael H Dahan	Canada	X		X	X
Robert Fischer	Germany		X	X	X
Peter Humaidan	Denmark	X		X	X
Antonio La Marca	Italy		X	X	X
Raoul Orvieto	Israel	X		X	X
Nikolaos P Polyzos	Spain/Belgium	X		X	X
Matheus Roque	Brazil	X		X	X
Sesh K Sunkara	UK		X	X	X
Filippo Maria Ubaldi	Italy		X	X	X
Lan Vuong	Vietnam	X		X	X
Hakan Yarali	Turkey		X	X	X
**Extended panel**					
Giuliano Bedoschi	Brazil	–	–	X	
Christophe Blockeel	Belgium	–	–	X	
Klaus Bühler	Germany	–	–	X	
Panagiotis Drakopoulos	Greece	–	–	X	
Juan José Espinó	Spain	–	–	X	
Shu Foong	Canada	–	–	X	
Michael Grynberg	France	–	–	X	
Shahar Kol	Israel	–	–	X	
Seang Lin Tan	Canada	–	–	X	
Juan Antonio Garcia-Velasco	Spain	–	–	X	
Joaquín Llace	Spain	–	–	X	
Le Long Ho	Vietnam	–	–	X	
Roy Homburg	Israel	–	–	X	
Sonia Malik	India	–	–	X	
Thi Minh Chau Le	Vietnam	–	–	X	
Dolors Manau	Spain	–	–	X	
Sezcan Mumusoglu	Turkey	–	–	X	
Evangelos Papanikolaou	Greece	–	–	X	
Margarida Silvestre	Portugal	–	–	X	
José Teixeira da Silva	Portugal	–	–	X	
Bülent Urman	Turkey	–	–	X	
Alberto Vaiarelli	Italy	–	–	X	
Amerigo Vitagliano	Italy	–	–	X	
Ariel Weissman	Israel	–	–	X	
Pedro Xavier	Portugal	–	–	X	

*Due to different time zones one web conference was held in the morning and a second in the afternoon

### The consensus process

**Fig. 1:**
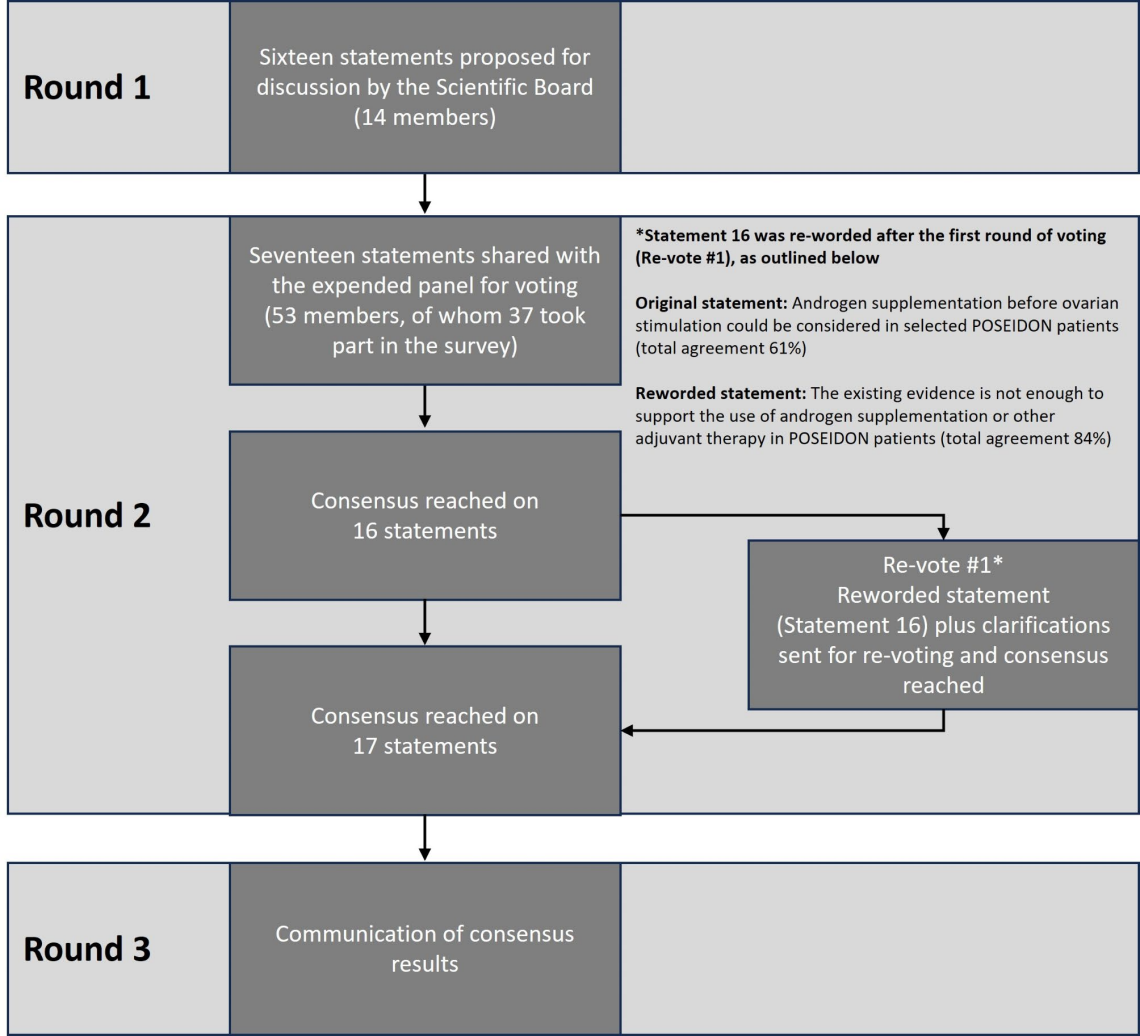
Overview of the Delphi consensus process and outcomes The extended panel voted on their level of agreement or disagreement with each of the 17 statements using a six-point Likert scale (1 = Absolutely agree; 2 = More than agree; 3 = Agree; 4 = Disagree; 5 = More than disagree; 6 = Absolutely disagree). Consensus was achieved if the proportion of participants either agreeing with a statement (responding “absolutely agree”, “more than agree” or “agree”) or disagreeing with a statement (responding “absolutely disagree” or “more than disagree” or “disagree”) exceeded 66% during Round 2. Statement 16 did not achieve consensus in Round 2; this was reworded, and the newly reworded statement was shared with the extended panel for voting on their level of agreement or disagreement. *One new statement was added during Round 1 following discussion

The Delphi consensus comprised three rounds (Fig. 1). During Round 1, statements and supporting references initially proposed by the two Scientific Coordinators were discussed and amended by the Scientific Coordinators and the 12 members of the Scientific Board during two web conferences (Table 1). The statements and references to be used in Round 2 were approved by the Scientific Board. During Round 2, an online survey was distributed to the 53 members of the extended panel. Of the 53 potential members of the extended panel, 37 joined the survey and 36 (68%) completed the entire survey and voted anonymously on their level of agreement with the statements using a six-point Likert-type scale (1 = Absolutely agree; 2 = More than agree; 3 = Agree; 4 = Disagree; 5 = More than disagree; 6 = Absolutely disagree). Consensus was achieved if the proportion of participants either agreeing with a statement (responding “absolutely agree”, “more than agree” or “agree”) or disagreeing with a statement (responding “absolutely disagree” or “more than disagree” or “disagree”) exceeded 66% during Round 2 [12, 13]. Statements that did not achieve consensus in Round 2 were discussed, revised and/or merged by the Scientific Board. The newly reworded statements were shared with the extended panel for voting on their level of agreement or disagreement. Participants were also asked to provide the main reason(s) for their response in an open-ended response field. Once consensus was achieved for all statements, WebEx conferences were arranged to communicate the outcome of Round 2 to the contributors (i.e., to report the level of agreement with each statement), to provide feedback to the participants and enable reflection on the statements (**Round 3**); attendance was not compulsory. Before discussing the results of the online survey, the previous stages of the Delphi consensus were briefly described, and the names of the Scientific Board members and the Scientific Panel were disclosed. The statements could not be amended after completion of Round 2.

## Results

### The Delphi process

**Fig. 2:**
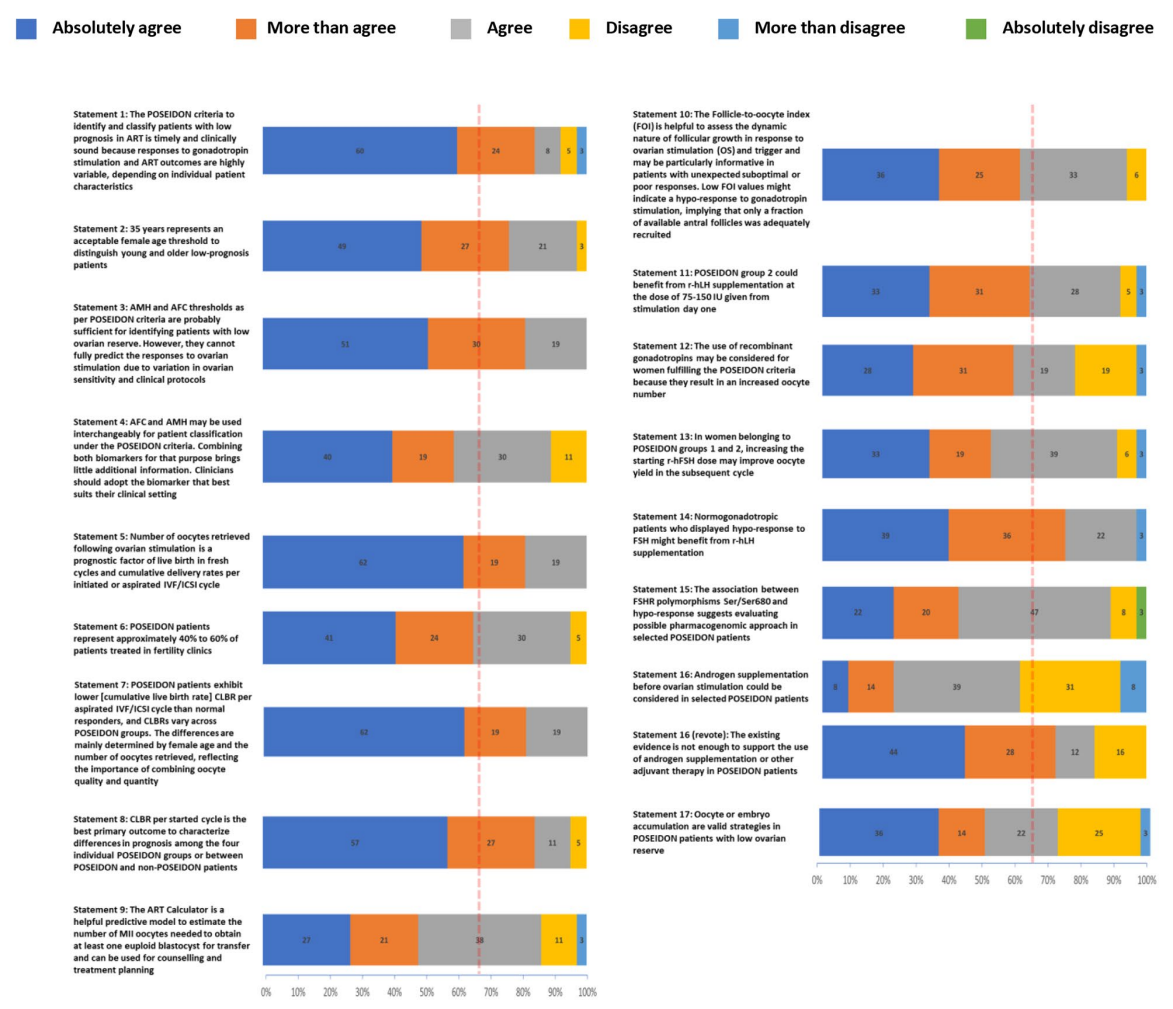
Results of voting on statements in Round 2 Results of the online survey distributed to the 53 members of the extended panel who voted anonymously on their level of agreement with the statements using a six-point Likert-type scale (1 = Absolutely agree; 2 = More than agree; 3 = Agree; 4 = Disagree; 5 = More than disagree; 6 = Absolutely disagree). Consensus was achieved if the proportion of participants either agreeing with a statement (responding “absolutely agree”, “more than agree” or “agree”) or disagreeing with a statement (responding “absolutely disagree” or “more than disagree” or “disagree”) exceeded 66% during Round 2 (red dotted line)

During Round 1, two of the initial 16 statements proposed by the Scientific Coordinators were approved without changes and 14 statements were approved after changes. One new statement (Statement 15) was included following discussion (Fig. 2).

During the first round of voting (Round 2), three statements (statements 3, 5 and 7) achieved 100% agreement, eight statements (statements 1, 2, 6, 8, 10, 11, 13 and 14) achieved > 90% agreement, three statements (statements 4, 9 and 15) achieved > 80% agreement, and two statements (statements 12 and 17) achieved > 70% agreement (Fig. 2). For statements 1, 9, 11, 12, 13, 14 and 17, 3% of the panel (*n* = 1 in each case) ‘more than disagreed’ with the statement; for one statement (the original wording of statement 16), 8% (*n* = 3) of the panel ‘more than disagreed’ with the statement. One member of the panel (3%) absolutely disagreed with statement 15. Statements with more than 20% disagreement (Statements 12 and 16) and the reasons for disagreement are shown in Supplementary Table 2. Statement 16 achieved only 61% agreement during the first round of voting. The statement was reworded and achieved 84% agreement after a second round of voting. Thirty-two of the 36 panel members voted for the inclusion of the new statement 15, which achieved a consensus agreement of 84%.

The agreed statements were subcategorized according to overall relevance, impact of female age, biomarkers’ relevance and thresholds, oocyte number, prevalence, low prognosis validation, primary outcome in POSEIDON studies, ART Calculator importance, role of the Follicle-to-Oocyte Ondex, management of POSEIDON Group 2, and management of POSEIDON patients.

### Statements

#### Statements relating to overall relevance

**Statement 1: The POSEIDON criteria to identify and classify patients with low prognosis in ART is timely and clinically sound because responses to gonadotropin stimulation and ART outcomes are highly variable**, **depending on individual patient characteristics.**

This statement received 92% agreement from the extended panel. Before POSEIDON, the ESHRE Bologna criteria for POR included a heterogeneous population and, therefore, could not support the routine use of any particular intervention for particular subsets of patients with POR, resulting in a lack of recommendations for the clinical handling of patients [4, 8]. The POSEIDON criteria introduced a more nuanced picture of the low prognosis patient in ART (Groups 1 & 2 supported the inclusion of variables that are not normally considered) and can guide the clinician to optimally manage patients with low prognosis through including a pragmatic endpoint for patient management and prediction of success (i.e., of the concept of oocyte quality) [4, 8, 9].

#### Statements relating to impact of female age

**Statement 2: 35 years represents an acceptable female age threshold to distinguish young and older low-prognosis patients.**

This statement achieved 97% agreement from the extended panel. Maternal age can impact oocyte and embryo competence through several mechanisms, including reduced ovarian reserve and decreased oocyte/embryo competence due to age-related chromosomal abnormalities (e.g., increased incidence of aneuploidies and possibly decreased mitochondrial activity, shortening of telomeres [14], cohesin dysfunctions, meiotic impairments during oogenesis, and flawed chromosome segregation patterns, such as non-disjunction, premature separation of sister chromatids, or reverse segregation), with 35 years defined as the lowest age threshold to define advanced maternal age (AMA) [15].

In a study reporting the results from chromosomal analysis performed on trophoectoderm biopsies of blastocysts, the lowest risk for embryonic aneuploidy was between ages 26 and 30. Both younger and older age groups had higher rates of aneuploidy and an increased risk for more complex aneuploidies. The overall risk did not measurably change after age 43 years [16].

In a retrospective study using logistic regression analysis of array comparative genomic hybridization (CGH) results of 7753 embryos from 990 patients, Ata et al. determined that increasing female age was associated with a significant decrease in euploidy rate in Day 3 and Day 5 embryos (*p* < 0.001 for both groups), and the probability of having at least one euploid embryo in an assisted cycle decreased by increasing female age (*p* < 0.01 for both Day 3 and Day 5 embryos) and was significantly increased by every additional embryo available (*p* < 0.01 for both Day 3 and Day 5 embryos) [6]. Equivalence of mean proportion of euploid versus aneuploid embryos (50%) was observed at age 35 years.

Furthermore, using a logistic regression model derived from the retrospective analysis of 1296 trophoectoderm biopsies (436 couples undergoing intracytoplasmic sperm injection [ICSI] and preimplantation genetic testing for aneuploidy) by next-generation sequencing (NGS), Esteves et al. reported on the age-related decrease in blastocyst euploid and the number of embryos needed to have at least one euploid blastocyst as a function of age [17]. This model predicted that the decrease in the probability of blastocyst euploidy followed an age-related binomial distribution. The geometric mean of the yearly variation was 13.6% and the decrease was progressive with every year of female age (decreasing by 1.2% at age 28, and by 2.0%, 3.5%, 6.7%, 9.8%, 13.6%, 17.9%, and 24.5% at ages 30, 32, 35, 37, 39, 41 and 44, respectively; *p* < 0.0001). The number of blastocysts required to obtain one euploid embryo began to increase progressively from the age of 35 years (from three at 28 years, to four at 35 years, and five, six, nine, sixteen and twenty nine for ages 37, 39, 41, 43, and 45, respectively) [10, 17].

Fecundity in women decreases gradually from the age of 32 years, with accelerated decrease after 37 years [18]. In light of the anticipated age-related decline in fertility, the increased incidence of disorders that impair fertility, and the higher risk of pregnancy loss, the American College of Obstetricians and Gynecologists and the American Society for Reproductive Medicine recommend that women older than 35 years should receive expedited evaluation and treatment within 6 months [19], whereas the joint committee of the American College of Obstetricians and Gynecologists and the American Society for Reproductive Medicine recommend women older than 40 years should receive immediate treatment and evaluation [18].

#### Statements relating to biomarkers’ relevance and thresholds

**Statement 3: AMH and AFC thresholds as per POSEIDON criteria are probably sufficient for identifying patients with low ovarian reserve. However**, **they cannot fully predict the responses to ovarian stimulation due to variation in ovarian sensitivity and clinical protocols.**

This statement achieved 100% agreement from the extended panel. AMH and AFC have a fundamental role in the prediction of POR in the POSEIDON criteria [20]. In their systematic review of tests predicting ovarian reserve and IVF outcome using receiver operating characteristic (ROC) analysis, Broekmans et al. concluded that ovarian reserve tests (including AMH) have only modest predictive value for poor response to hyperstimulation, whereas AFC could be considered as a clinically adequate test for poor response prediction at a low threshold level in normal cycling women [21]. However, in a meta-analysis of 13 studies reporting on AMH and 17 studies reporting on AFC, the ROC curves for poor response and non-pregnancy showed no significant difference between the two markers, leading the authors to conclude that AMH has at least the same level of accuracy and clinical value for the prediction of poor response and non-pregnancy as AFC [22]. Nevertheless, they acknowledged that clinical applicability ultimately depends on the way that abnormal test results might alter patient management. A more recent systematic literature review (2013) demonstrated that AFC (41 studies) and AMH (25 studies) were the most sensitive markers of ovarian reserve at the time of the review and were pivotal in planning personalized ovarian stimulation protocols (based on the selective use of gonadotropin hormone-releasing hormone [GnRH] analogs and gonadotropin starting dose adjustment), owing to their reliable accuracy across the extremes of ovarian response and their interchangeability [23].

Notably, the above analyses predate the development of the POSEIDON criteria and, accordingly, AMH and AFC were investigated in isolation in these studies. By contrast, the POSEIDON criteria consider these in the context of the age of the patient, previous response to ovarian stimulation and other risk factors for POR, providing a substantial advancement in the diagnosis and clinical management of these patients, although without relevant improvements in IVF outcomes [20]. A population-based cohort study of 9484 consecutive patients treated at three fertility centres between 2015 and 2017, assessed the agreement between AFC and AMH levels in the context of patient classification using the POSEIDON criteria. The study showed that for low oocyte yield, the optimal AFC and AMH cutoff values were five and 1.27 ng/ml, with sensitivities of 0.61 and 0.66, specificities of 0.81 and 0.72, and AUC receiver operating characteristics of 0.791 and 0.751, respectively, which were similar to the thresholds included in the Poseidon criteria. However, owing to the low predictive value of POSEIDON for low oocyte yield, the authors recommended that clinicians should adopt the biomarker that is most reflective of each clinical setting [24]. Furthermore, in the development of the concept of Follicle-to-Oocyte Index (FOI), Alviggi et al. defined these biomarkers as a static snapshot of an individual’s ovarian reserve that is not reflective of the dynamic nature of follicular growth in response to exogenous ovarian stimulation when used in isolation [25].

**Statement 4: AFC and AMH may be used interchangeably for patient classification under the POSEIDON criteria. Combining both biomarkers for that purpose brings little additional information. Clinicians should adopt the biomarker that best suits their clinical setting.**

This statement achieved 89% agreement from the extended panel. In a real-world study of 9484 consecutive patients by Esteves et al., the degree of agreement in classifying patients according to POSEIDON groups was strong overall (kappa = 0.802; 95% CI 0.792; 0.811). Furthermore, the study showed that for low oocyte yield, the optimal AFC and AMH cutoff values were five and 1.27 ng/ml, with sensitivities of 0.61 and 0.66, specificities of 0.81 and 0.72, and AUC receiver operating characteristics of 0.791 and 0.751, respectively, which were similar to the thresholds included in the POSEIDON criteria [24].

#### Statements relating to oocyte number

**Statement 5: Number of oocytes retrieved following ovarian stimulation is a prognostic factor of live birth in fresh cycles and cumulative delivery rates per initiated or aspirated IVF/ICSI cycle.**

This statement achieved 100% agreement from the extended panel. When utilizing all fresh and frozen embryos in 1099 women undergoing their first ovarian stimulation cycles followed by single embryo transfer (ET) according to ovarian response category (poor: 1–3 oocytes; suboptimal: 4–9 oocytes; normal: 10–15 oocytes; high: >15 oocytes), low responders had a lower live birth rate (LBR) in fresh transfer cycles compared with suboptimal, normal and high responders (*p* < 0.05) and multivariable logistic regression analysis showed this was an independent predictive factor (*p* < 0.001) for CLBR [26]. In another retrospective multicenter analysis of individual patient data in 14,469 patients undergoing their first cycle of IVF/ICSI between 2009 and 2014, CLBR steadily increased with the number of oocytes, reaching 70% when ≥ 25 oocytes were retrieved and more modest increases above 27 oocytes; live-birth probability in fresh transfer cycles increased up to seven oocytes and then levelled off between seven and 25 oocytes, and decreased thereafter, which could be attributed to an increase in freeze-all cycle rate in patients with > 20 oocytes retrieved [27]. Notably, maximum CLBR was observed up to 25 oocytes in a large retrospective study of 221,221 autologous cycles between January 2009 and December 2015 but only in women aged between 18 and 35 years, showing that the maximum CLBR observed during ART is dependent on female age. In older women (36–44 years) the maximum CLBR was achieved beyond 30 oocytes, dropping to nine oocytes in women ≥ 45 years [28].

Furthermore, in a systematic review and meta-analysis, which included the study by Drakopoulos and Blockeel, 28 studies (three prospective and 25 retrospective) reporting on 291,752 ART cycles confirmed a positive correlation between oocyte number and the number of top/good quality embryos (*p* < 0.001 for correlations with Day 2/3 embryos, Day 5/6 embryos metaphase II [MII] oocytes, oocytes with two pronuclei and euploid embryos), suggesting that increased oocyte yield from a single cycle stimulated cycle may maximize outcomes [29].

#### Statements relating to prevalence

**Statement 6: POSEIDON patients represent approximately 40–60% of patients treated in fertility clinics.**

This statement achieved 95% agreement from the extended panel. Of 13,146 patients included in a multicentre population-based cohort study in fertility clinics in Brazil, Turkey, and Vietnam, POSEIDON patients represented 43.0% (95% confidence interval [CI] 42.0–43.7) of the studied population, and the prevalence rates varied across study centers (range: 38.6–55.7%) [30]. However, this range may be an over- or under-estimation, and more data are needed, particularly from Europe and the USA, to gain insight into the true number. For example, in a retrospective cohort study of 62,749 women (97,388 cycles) who underwent ART treatment at the Reproductive and Genetic Hospital of CITIC-XIANGYA, China, between January 2014 and June 2017, 19,781 (31.52%) women fulfilled the POSEIDON criteria (26,697 cycles) [31]. Lastly, of a total of 32,128 fresh IVF cycles from January 2014 to October 2018 at a single IVF clinic in Xi’an, China, 6383 (19.9%) were low prognosis, based on the POSEIDON criteria [32].

#### Statements relating to low prognosis validation

**Statement 7: POSEIDON patients exhibit lower [cumulative live birth rate] CLBR per aspirated IVF/ICSI cycle than normal responders**, **and CLBRs vary across POSEIDON groups. The differences are mainly determined by female age and the number of oocytes retrieved**, **reflecting the importance of combining oocyte quality and quantity.**

This statement received 100% agreement from the extended panel. In a multicenter population-based retrospective cohort study involving 9073 patients treated in three fertility clinics in Brazil, Turkey and Vietnam between 2015 and 2017, the CLBR in POSEIDON patients was between 33.7% vs. 50.6% lower than in normal responders (*p* < 0.001) and varied across POSEIDON groups (Group 1 [*n* = 212] 27.8%; Group 1b [*n* = 1785] 47.8%; Group 2a [*n* = 293] 14.0%; Group 2b [*n* = 1275] 30.5%; group 3 [*n* = 245] 29.4%; Group 4 [*n* = 623] 12.5%). In POSEIDON Groups 1 and 2, the CLBR was twice as high in suboptimal responders (4–9 oocytes retrieved) than in poor responders (< 4 oocytes retrieved) (*p* = 0.0004). Predictors of CLBR as ascertained using logistic regression analysis were POSEIDON grouping, number of embryos obtained, number of ET cycles per patient, number of oocytes collected, female age, duration of infertility and body mass index (*p* < 0.001) [33].

In a retrospective cohort study of 62,749 women (97,388 cycles) undergoing ART treatment at the Reproductive and Genetic Hospital of CITIC-XIANGYA between January 2014 and June 2017, > 30% of women undergoing IVF/ICSI may be classified as low prognosis. Different reproductive outcomes were observed among the four POSEIDON groups; after three successive cycles of treatment, the most optimal outcomes were observed in Groups 1, 2 and 3. The variables associated with live birth in the first cycle were POSEIDON stratification and ovarian stimulation protocol; for the second stimulation cycle, POSEIDON stratification (except Group 3) and ovarian stimulation protocol were associated with live birth [31]. In another retrospective cohort study of 10,615 women who underwent IVF treatment at the Peking University between January and December 2017, the CLBR in the first cycle in each of the POSEIDON groups was lower (*p* > 0.001) than in non-POSEIDON patients. In the second cycle, CLBR was lower in older patients (Groups 2b [*p* = 0.001] and 4 [*p* < 0.001]) and in younger patients with poor ovarian response (Group 3 [*p* = 0.019]) compared with non-POSEIDON patients; younger patients had higher CLBR than older patients in both cycles (*p* < 0.001) [34]. Furthermore, data from a multicenter observational cohort study of 551 low-prognosis women age < 44 years initiating IVF/ICSI treatment with fixed-dose 150 IU/day follicle-stimulating hormone (FSH) between 2011 and 2014 reported a mean CLBR of 56% over 18 months of treatment. CLBR varied among POSEIDON groups, primarily determined by age (younger unexpected poor responder ∼ 65%, younger unexpected suboptimal responders ∼ 68%, younger expected poor responders ∼ 59%, older unexpected poor responders 42%, older unexpected suboptimal responders 54%, older expected poor responders ∼ 39%) compared with younger normal responders (∼ 72%) and older normal responders (∼ 58%) [35]. Finally, using statistical modelling, a retrospective cohort study conducted at McGill University Health Center on 459 patients who underwent IVF treatment between 2011 and 2014 showed that age and ovarian response were both predictors of pregnancy and live birth in women with poor response (one or two follicles ≥ 14 mm) and grouped according to AFC (0–5, 6–10 and ≥ 11), in concert with the POSEIDON criteria classifications. The likelihood of live birth as a function of age and AFC showed that a 1-year increase in age reduces the likelihood of live birth by 11%, whereas a one unit increase in AFC lead to a 9% increase in the odds of a live birth (*p* < 0.05 for both). AFC had a significant effect in the youngest age group: women with AFC > 11 had a 56% LBR compared with 6% LBR in those with AFC ≤ 11 [36].

#### Statements relating to primary outcome in POSEIDON studies

**Statement 8: CLBR per started ovarian stimulation cycle is the best primary outcome to characterize differences in prognosis among the four individual POSEIDON groups or between POSEIDON and non-POSEIDON patients.**

This statement received 95% agreement from the extended panel. A conservative estimate of a couple’s chance of a live birth over an entire treatment course (CLBR) was reported as 51% (95% CI 49–52) after six cycles in 614 patients (14,248 cycles); the optimistic estimate was 72% (95% CI 70–74), with higher rates in those aged < 35 years than in those aged ≥ 40 years. CLBR declined with increasing age, and age-stratified curves for women < 35 years versus ≥ 40 years were significantly different (*p* < 0.001) [37].

In their literature review, Moragianni et al. (2010) emphasized the need to highlight CLBR during counselling for couples with infertility as a more realistic estimate of success, when taking maternal age and genetic factors into consideration [38]. This point was reiterated by Maheshwari et al. (2015) in their later review of the literature. They recommended that CLBR is generally perceived to be the preferred reporting system in IVF and called for an international consensus on how this is calculated, reported and interpreted across the world [39]. CLBR was further defined in the International Glossary on Infertility and Fertility Care (2017) as ‘The number of deliveries with at least one live birth resulting from one initiated or aspirated ART cycle, including all cycles in which fresh and/or frozen embryos are transferred, until one delivery with a live birth occurs or until all embryos are used, whichever occurs first’ [40] and in the ESHRE guideline: ovarian stimulation for IVF/ICSI (2020) [41], which provided guidelines for efficacy in terms of CLBR (or LBR) per cycle. Using these criteria in their multicenter population-based retrospective cohort study of 9073 patients, Esteves et al. reported that the CLBR of POSEIDON patients was on average 50% lower than in normal responders and varied across POSEIDON groups. The differences were primarily determined by female age, number of embryos obtained, number of ET cycles per patient, number of oocytes retrieved, duration of infertility, and BMI [33].

Notably, although CLBR is recognized as a suitable way to report the success of IVF treatment, calculation of this outcome varies between studies. While some studies report CLBR as all live births per the number of women who attempted stimulation, CLBR has also been reported as at least one live birth episode per the number of women who had oocyte collection. New recommendations consider the reporting of CLBR in the short term (first live birth per women in the 2 years after one oocyte retrieval), medium term (all live births per women in the 5 years after one oocyte retrieval) and long term (all live births per women over 10 years after three oocyte retrievals) [39].

#### Statements relating to ART Calculator importance

**Statement 9: The ART Calculator is a helpful predictive model to estimate the number of MII oocytes needed to obtain at least one euploid blastocyst for transfer and can be used for counselling and treatment planning.**

This statement received 86% agreement from the extended panel. Maternal age can impact oocyte and embryo competence through several mechanisms, including reduced ovarian reserve and decreased oocyte/embryo competence due to aging-related chromosomal abnormalities (as discussed in Statement 2) [15]. Using a logistic regression predictive model derived from the retrospective analysis of 1296 trophoectoderm biopsies (436 couples undergoing ICSI and preimplantation genetic testing for aneuploidy) by next-generation sequencing the probability of blastocyst euploidy decreased according to an age-related binomial distribution, progressing with each additional year (from 1.2% at age 28 years to 24.5% at age 44 years; *p* < 0.0001) [17]. The number of blastocysts required to obtain one euploid embryo began to increase progressively from the age of 35 years (from three at 28 years, to four at 35 years); therefore, more oocytes and embryos will be needed with increasing maternal age to counteract this decrease.

In a randomized controlled trial (RCT) of 205 infertile couples with a female partner < 43 years and with AMH ≥ 1.2 ng/mL and Day 3 FSH < 12 IU/L/, Forman et al. reported that during embryo selection using comprehensive chromosome screening, the transfer of a single euploid blastocyst results in ongoing pregnancy rates that are the same as transferring two untested blastocysts while dramatically reducing the risk of twins [42]. However, while PGT-A may reduce the time to live birth by decreasing the risk of pregnancy loss in certain populations, the available evidence is insufficient to support the use of preimplantation genetic testing for aneuploidy in routine clinical practice [43, 44].

To estimate the number of oocytes needed to achieve at least one euploid embryo for transfer and provide a revised estimate when fewer than the predicted number of embryos were obtained after one or more oocyte retrieval cycle, Esteves et al. assessed the factors that influenced embryo ploidy and estimated the predicted probability of blastocyst euploidy as a function of each mature oocyte retrieved [10]. Using a negative binomial distribution to model the number of euploid blastocysts and the adaptive LASSO (Least Absolute Shrinkage and Selection Operator) method for variable selection, the fitted model identified female age, sperm source used for ICSI, and the number of mature (metaphase II) oocytes as predictive factors (*p* < 0.0001). In the final predictive model, developed using logistic regression analysis, and internally validated by the holdout method, the estimated predicted probabilities of a mature oocyte developing into a euploid blastocyst decreased progressively with female age and was negatively modulated overall by use of testicular sperm across age (*p* < 0.001) [10].

Using this model, the ART Calculator was developed to make two types of predictions automatically: pretreatment information to estimate the minimum number of oocytes to achieve ≥ 1 euploid blastocysts; and pretreatment information and the actual number of mature oocytes collected or accumulated to provide a revised estimate of the probability of achieving the aforesaid outcome when fewer than the predicted number of mature oocytes are obtained after one or more oocyte retrieval cycles [10]. The ART Calculator was validated in a multicenter study using retrospective clinical and embryonic data from 1464 consecutive infertile couples who had IVF/ICSI with the intention to have preimplantation genetic testing for aneuploidy. This analysis showed that the fitting between the ART Calculator and the validation model were sufficiently close for both the estimated probabilities of a euploid blastocyst per MII oocyte (*r* = 0.91) and the minimum number of MII oocytes (*r* = 0.88). The frequency of patients with at least one euploid blastocyst among those who achieved the estimated minimum number of MII oocytes were 84.8% (70% predicted probability of success), 87.5% (80% predicted probability of success) and 90% (90% predicted probability of success) [11].

Recently, the ART Calculator was rebranded as ‘ONE – Oocyte Number Estimator’ and is freely available online (https://art-one.merckgroup.com/art) for professionals and patients) [45].

#### Statements relating to the Follicle-to-Oocyte Index

**Statement 10: The Follicle-to-OocyteIndex (FOI) is helpful to assess the dynamic nature of follicular growth in response to ovarian stimulation (OS) and trigger and may be particularly informative in patients with unexpected suboptimal or poor responses. Low FOI values might indicate a hypo-response to gonadotropin stimulation**, **implying that only a fraction of available antral follicles was adequately recruited.**

This statement received 94% agreement among the extended panel. The FOI, the ratio between the number of oocytes collected at ovum pick up and the number of antral follicles at the beginning of ovarian stimulation, expressed as a range from 0 to 100, was proposed by Alviggi et al. as a novel parameter to assess hypo-response. The value of FOI compared with traditional ovarian reserve markers is that it might optimally reflect the dynamic nature of follicular growth in response to exogenous gonadotropins in light of the many non-mutually exclusive factors that influence ovarian resistance to ovarian stimulation (low gonadotropin starting dose, genetic or environmental factors, asynchronous follicle development, timing and mode of final follicular maturation trigger, and technical issues related to oocyte retrieval) [25].

Importantly, genotype is implicated in the outcome of ovarian stimulation, as reported in a systematic review and meta-analysis of 33 studies, in which ovarian stimulation outcomes related to seven polymorphisms (FSH receptor [FSHR; rs6165, FSHR rs6166, FSHR rs1394205]; luteinizing hormone beta subunit [LHB; rs1800447, LHB rs1056917]; luteinizing hormone chorionic gonadotropin receptor [LHCGR; rs2293275] and LHCGR [rs13405728]) were evaluated. Higher FSH consumption is expected in homozygotes for the A allele of the FSHR (rs1394205) polymorphism than carriers of the G allele. Moreover, FSHR (rs6166) GG homozygotes have fewer oocytes than AA and AG carriers. It is feasible that the effect of these polymorphisms on ovarian stimulation may partially explain the phenomenon of ‘hypo-response’ [46–49].

To investigate ovarian sensitivity in subgroups of patients with low prognosis, as defined by the POSEIDON criteria, using FOI and the follicle output rate (FORT), Chen et al. performed a retrospective cohort study of 32,128 treatment cycles from a single IVF clinic between January 2014 and October 2018. Using FORT as a marker for ovarian sensitivity, this was highest in POSEIDON Group 3, followed by Group 4, Group 1 and Group 2, and the trend in FOI values was consistent with those for FORT. Adjustment of the ovarian stimulation protocol was recommended for patients with poor ovarian sensitivity, whereas an adjustment to the gonadotropin starting dose was preferred for those with normal ovarian sensitivity [32]. Furthermore, adjustment of time and mode of final follicular maturation trigger may be of benefit for patients with normal FORT and low FOI [50].

Finally, a retrospective analysis of 264 IVF cycles after the first and cumulative ETs investigated the relation between oocyte yield (total retrieved oocytes [Oc] and total mature oocytes ([MII]) relative to the antral follicle count (Oc/AFC and MII/AFC) and AMH (Oc/AMH and MII/AMH). Oc/AMH and MII/AMH ratios had no effect on the occurrence of LBR or on implantation rate after the first or cumulative ET when stratified by age < 36 years and > 39 years, whereas the ratios of Oc/AFC and MII/AFC seemed promising indicators to assess ovarian response [51].

#### Statements related to management of POSEIDON group 2

**Statement 11: POSEIDON Group 2 could benefit from r-hLH supplementation at the dose of 75–150 IU given from stimulation day one.**

This statement achieved 92% agreement by the extended panel. A single-center randomized, parallel group, comparative study aimed to identify potential benefits of mid-follicular (from Day 6) r-hLH supplementation in 131 women (68 allocated to recombinant human FSH [r-hFSH] and 63 allocated to r-hFSH plus r-hLH) aged 35–39 years undergoing ovarian stimulation for ICSI. No differences were observed in oocyte or embryo quality or quantity; however, higher implantation rates (11.3% vs. 18.1%; *p* = 0.049) and live births per started cycle (7.4 vs. 19.0; *p* = 0.047) were observed with r-hLH supplementation [52]. Similarly, in a randomized open-label controlled trial of r-hFSH versus r-hFSH plus r-hLH from Day 1 in two age groups (≤ 35 years [380 women] and 36–39 years [340 women]), implantation rates were significantly higher in the older age group who received r-hLH compared with those who received r-hFSH alone (odds ratio [OR] 1.56, 95% CI 1.04–2.33), although there was no difference in ongoing pregnancy per cycle (OR 1.49, 95% CI 0.93–2.38) and no effect on either outcome in younger women (implantation rate: OR 1.03, 95% CI 0.73–1.47; ongoing pregnancy rate: OR 1.0, 95% CI 0.66–1.52) [53]. Furthermore, in a systematic review and meta-analysis of 12 RCTs of women aged 35–40 years, r-hFSH/r-hLH cotreatment was associated with higher implantation rates (OR 1.49, 95% CI 1.10–2.01) and clinical pregnancy rates (OR 1.45, 95% CI1.05–2.00) compared with r-hFSH monotherapy, despite fewer oocytes being retrieved in the r-hFSH/r-hLH group than in the r-hFSH group [54].

Lastly, the timing of LH supplementation during ovarian stimulation was assessed in an open-label study of women aged 36–40 years who received r-hFSH plus r-hLH from simulation Day 1 (*n* = 103) compared with those who received r-hFSH alone for Days 1–5 followed by r-hFSH plus r-hLH from Day 6 (*n* = 99). Although the difference in number of oocytes retrieved between groups (–1.28, 95% CI -3.15 to 0.59) did not reach the predefined limit of equivalence (± 3 oocytes), the implantation rate (24.7% vs. 13.3%) and clinical pregnancy rate per started cycle (31.6% vs. 17.2%) and per ET (34.4% vs. 18.9%) were numerically higher in the women who received r-hLH from Day 1 compared with those who received r-hLH from Day 6, suggesting that the potential for initiating LH supplementation earlier during the ovarian cycle warranted further investigation [55].

#### Statements related to management of POSEIDON patients

**Statement 12: The use of recombinant gonadotropins may be considered for women fulfilling the POSEIDON criteria because they result in an increased oocyte number.**

This statement achieved 78% agreement by the extended panel. According to ESHRE 2019 guidelines on controlled ovarian stimulation, there is insufficient valid scientific evidence to favor the use of one type of gonadotropin over another in POR [56]. However, favorable outcomes have been reported with r-hFSH in several studies. In a randomized, open-label, assessor-blinded multinational study, women with anovulatory infertility were randomized to stimulation with highly purified human menopausal gonadotropin (HP-hMG; *n* = 91) or r-hFSH (*N* = 93). Although non-inferiority in ovulation rate was reported between treatments (85.7% vs. 85.5%), significantly fewer intermediate-sized follicles (12–16 mm) were observed in the HP-hMG group (*p* < 0.05) [57]. In an open-label prospective randomized comparison including 629 women at 18 Dutch IVF centers who received a fixed dose of 150 IU/day HP-hMG or r-hFSH, treatment with HP-hMG resulted in statistically significantly fewer oocytes (*n* = 7.8) than treatment with r-hFSH (*n* = 10.6; *p* < 0.0001) [58]. Similarly, a meta-analysis of 16 RCTs reporting on hMG and r-hFSH preparations (4040 patients) found that treatment with hMG resulted in fewer oocytes compared with r-hFSH preparations in the unadjusted analysis (-1.54, 95% CI -2.53 to -0.56; *p* < 0.0001) and the analysis adjusted for baseline characteristics (-2.10, 95% CI -2.83 to -1.36; *p* < 0.001), despite a higher mean total dose of hMG compared with r-hFSH (standardized mean difference, 0.33 [95% CI: 0.08 to 0.58; *P* = 0.01]) [59]. Lastly, a randomized, open-label, assessor-blind, parallel groups, multicenter, noninferiority trial of 749 women in 27 infertility centers in seven countries assessed controlled ovarian stimulation (COS) with HP-hMG or r-hFSH in a GnRH antagonist cycle. Participants had a compulsory single-blastocyst transfer on Day 5 in one fresh or subsequent frozen blastocyst replacement in natural cycles initiated within 1 year of each patient’s start of treatment. The number of follicles ≥ 12 mm on Day 6 of stimulation were 4.2 ± 3.1 in the r-hFSH group compared with 3.6 ± 3.8 in the HP-hMG group (*p* = 0.011). At the end of stimulation, the number of follicles ≥ 12 mm was 10.9 ± 4.7 versus 11.8 ± 4.9 (*p* = 0.25) and the number 12–14 mm was 3.8 ± 2.9 versus 3.3 ± 2.6 (*p* = 0.024). Non-inferiority between r-hFSH and HP-hMG was also established for ongoing pregnancy rate [60]. With regard to treatment with r-hFSH, in the ESPART trial 939 women classified as POR according to a criteria incorporating the ESHRE Bologna Criteria) were randomized to treatment with r-hFSH:r-hFSH (*n* = 477) or r-hFSH alone (*n* = 462) [61]. The mean (SD) number of oocytes retrieved (primary endpoint) was higher in the r-hFSH only group (3.6 [2.82]) compared with the r-hFSH:r-hLH group (3.3 [2.71]), and the mean (SD) number of MII oocytes in ICSI patients (secondary outcome) was also higher in the r-hFSH group (3.1 [2.14]) compared with the r-hFSH only group (2.9 [2.07]), although the between-group differences were not statistically significant.

**Statement 13: In women belonging to POSEIDON groups 1 and 2**, **increasing the starting r-hFSH dose may improve oocyte yield in the subsequent cycle.**

This statement achieved 91% agreement by the extended panel. In a longitudinal study of 160 women treated in their second cycle with either the same (*n* = 53) or a higher (*n* = 107) starting dose of r-hFSH than in the first cycle, a significantly higher number of oocytes was retrieved in the second cycle (6 [5–8] vs. 9 [6–12]; *p* < 0.001) in the increased dose group. After conducting a generalized estimating equation multivariable regression analysis, while adjusting for relevant confounders, the dose increment of r-hFSH was the only significant predictor of the number of oocytes retrieved in subsequent cycles (regression coefficient 0.02; *p* = 0.007), implying that an increase of 50 IU of the initial dose would lead to one more oocyte [62].

In a review of the literature, Conforti et al. reiterated that a polygenic trait involving gonadotropins and/or their receptors seems to be the primary pathophysiological mechanism explaining this phenomenon. Among currently existing pharmacological interventions, use of r-hFSH in preference to urinary gonadotropin preparations, FSH dosage increase, and use of r-hLH supplementation may be considered, alone or in combination, for optimally managing POSEIDON Groups 1 and 2 patients [63].

**Statement 14: Normogonadotropic patients who displayed hypo-response to FSH might benefit from r-hLH supplementation.**

This statement achieved 97% agreement from the extended panel. In a prospective randomized study, women showing hypo-responsiveness to FSH were randomized to receive an increased dose of FSH (*n* = 54), coadministration of r-hLH and an increased dose of FSH (*n* = 54), administration of FSH and LH activity as hMG (*n* = 22) or FSH with no increase (*n* = 54; control group). Implantation rates and pregnancy rates per ET were higher in those with r-hLH supplementation compared with those with increased FSH dose or those receiving hMG (*p* < 0.05 for all comparisons). The LBR was similar between the r-hLH supplemented patients and the control group (40.7% and 37%, respectively) and was twice the rate of the increased FSH group (22%) and the hMG group (18%) [64]. In a multicenter, prospective, RCT in 260 normogonadotropic women, the effect of supplementation with r-hLH (*n* = 65) was compared with a 150 IU step-up of r-hFSH starting dose (225 IU) from Day 8 (*n* = 65) or no change in the starting dose (*n* = 130). The mean number of cumulus–oocyte complexes retrieved in the r-hLH group (9.0 ± 4.3) was significantly higher (*p* < 0.01) compared with the r-hFSH step-up group (r-FSH 6.1 ± 2.6), but significantly lower than in those with no change in the starting dose (10.49 ± 3.7, *P* < 0.05). Implantation and pregnancy rates were significantly lower (*p* < 0.05) in the r-hFSH step-up group (10.5 and 29.3% respectively) when compared with normal responders (18.1 and 47.3% respectively). The authors concluded that r-hLH supplementation was more effective than increasing the dose of r-hFSH in terms of ovarian outcome in patients with an initial inadequate ovarian response to r-FSH alone [65]. In a retrospective, single-center, cohort study of 65 women with AMH > 0.5 ng/ml and/or AFC > 5 and poor ovarian response in their first cycle, treatment with the same starting dose of r-FSH used in the first cycle plus daily addition of 150 IU of r-LH from Day 1 resulted in an increase in number of oocytes retrieved (4.0 ± 3.1 vs. 2.7 ± 1.8; *p* < 0.001), number of metaphase II oocytes in cycles where ICSI was considered (2.8 ± 2.7 vs. 2.1 ± 1.6; *p* < 0.04), E2 levels at hCG triggering (1358 ± 851 vs. 895 ± 560; *p* < 0.001) and mean (SD) number of embryos transferred compared with the first cycle (1.3 ± 1.1 vs. 0.8 ± 0.9; *p* = 0.002). A 15% clinical pregnancy rate (10 pregnancies, including two twin pregnancies) was also observed in the second cycle [66]. Finally, in a recent, non-interventional study of real-world data from the Deutsches IVF-Register (D·I·R), Bielfeld et al. compared the effectiveness of r-hFSH and r-hLH in a 2:1 ratio (*n* = 4,250 women) versus r-hFSH alone (*n* = 10,236 women) for ovarian stimulation during ART treatment. In a post-hoc analysis using propensity score matching, in women with normal ovarian reserve (5–14 oocytes) aged 35–40 years the treatment effect was significantly higher for r-hFSH:r-hLH (*n* = 2283) compared with r-hFSH alone (*n* = 2517) in terms of clinical pregnancy (33.1% [95% CI 31.0, 35.0] vs. 28.5% [26.6, 30.4]; *P* = 0.001, not adjusted for multiplicity) and live birth (22.5% [20.5, 24.2] vs. 19.4% [17.6, 20.9]; *P* = 0.014, not adjusted for multiplicity), highlighting the potential benefits of r-hFSH:r-hLH for OS in women aged 35–40 years with normal ovarian reserve [67].

The clinical efficacy of r-hLH supplementation was evaluated in a study of patients with a suboptimal response to ovarian stimulation undergoing assisted reproduction with GnRH-agonist downregulation and stimulation with r-hFSH. One hundred and thirty-seven patients were included in the study, of whom 52 showed normal ovarian response and comprised the control group (Group 1); the remaining suboptimal responders were divided into Groups 2 (75 IU/L r-hLH added to r-hFSH; *n* = 50) and 3 (r-hFSH dose was increased by 75 IU/L; *n* = 35). Implantation rates were significantly higher in Groups 1 and 2 compared with Group 3 (34.8%, 36.1% and 15.0%, respectively; *p* < 0.02). Pregnancy rates were numerically higher in Groups 1 and 2 (64.7% and 57.8%, respectively) compared with Group 3 (32.4%), and significantly higher in Group 2 versus Group 3 (*p* < 0.05) [68]. Additionally, a systematic review and meta-analysis of prospective clinical trials in which recombinant FSH monotherapy protocols were compared with LH-supplemented protocols in hypo-responders, found that significantly higher oocyte numbers (weighted mean differences 1.98; *p* = 0.03), implantation rates (OR 2.64; *p* = 0.004) and clinical pregnancy rates (OR 2.03; *p* = 0.003) were observed in hypo-responders supplemented with r-hLH versus hypo-responders who underwent FSH monotherapy [69].

Lastly, a retrospective, real-world analysis of 1470 women with poor, suboptimal or normal response to COS undergoing IVF with either r-hFSH alone or r-hFSH plus r-hLH, reported a significantly higher CLBR in patients receiving r-hFSH alone compared with those receiving supplementation with r-hLH (29.3 vs. 22.2%, respectively; *p* < 0.01). However, when only poor and suboptimal responders were considered, comparable CLBRs were observed (15.6 vs. 15.2%, *p* = 0.95), despite those receiving r-hFSH plus r-hLH having a significantly higher mean age (38.3 ± 3.5 years vs. 36.4 ± 4.3 years, *p* < 0.01) and poorer ovarian reserve markers. The authors concluded that r-hLH supplementation in COS may represent a reasonable option for patients with predictable or unexpected poor or suboptimal ovarian response to r-hFSH, those matching the ESHRE Bologna criteria for poor responsiveness, and those included in the POSEIDON classification [70]. In summary, given the wealth of clinical evidence to date, r-hLH supplementation is an option for the management of women belonging to POSEIDON Group 1, especially in cases of genetic variations of the LH gene [71].

It is important to consider the timing of r-hLH supplementation to avoid losing the window of opportunity during folliculogenesis, with early supplementation showing a potential benefit. An open-label, equivalence study compared r-hFSH plus r-hLH from stimulation Day 1 (Group A; *n* = 103) with r-hFSH alone on stimulation Days 1–5, followed by r-hFSH plus r-hLH (2:1 ratio) from stimulation Day 6 (Group B; *n* = 99) in women aged 36–40 years undergoing ovarian stimulation for ART. The mean (± standard deviation) number of oocytes retrieved was 9.7 (± 6.9) and 10.9 (± 6.5) in Group A and B, respectively; the estimated difference between groups (− 1.28 oocytes [95% CI: −3.15 to 0.59]) did not reach the predefined limit of equivalence (± 3 oocytes) and, therefore, the primary objective was not met. However, the authors concluded that the potential benefit of initiating LH supplementation from Day 1 of ovarian stimulation is likely superior to a later rLH addition to rFSH on Day 6, as higher implantation rates and clinical pregnancy rates were obtained when rFSH-rLH combination treatment was started from Day 1 in women aged ≥ 35 years [55].

**Statement 15: The association between FSHR polymorphisms Ser/Ser680 and hypo-response suggests evaluating possible pharmacogenomic approach in selected POSEIDON patients.**

This statement achieved 89% agreement from the extended panel. A prospective, randomized, controlled study evaluated whether the same daily dose of FSH resulted in lower levels of estradiol in women homozygous for the p.N680S FSH receptor sequence variation, and whether the difference could be overcome by higher FSH doses. Women undergoing COS for IVF or ICSI, and who were homozygous for the wild-type or for p.N680S, were randomly assigned to Group I (Ser/Ser; *n* = 24), receiving a FSH dose of 150 IU/day; Group II (Ser/Ser; *n* = 25), receiving a FSH dose of 225 IU/day; or Group III (Asn/Asn; *n* = 44) received a FSH dose of 150 IU/day. Age and basal FSH levels were comparable between groups. At ovulation induction, total FSH doses were comparable in Group I (1631 ± 96 IU) and Group III (1640 ± 57 IU), but significantly higher in Group II (2421 ± 112 IU) (*p* < 0.001). Peak estradiol levels on the day of hCG administration were significantly lower in Group I (5680 ± 675 pmol/L) compared with Group III (8679 ± 804 pmol/L) (*p* = 0.028) and increasing the FSH dose from 150 to 225 IU/day overcame the lower estradiol response in women with Ser/Ser (Group II, 7804 ± 983 pmol/L) [72].

A prospective study of 124 patients aged 24–41 years who had undergone COS for IVF-ET evaluated whether carriers of common single nucleotide polymorphisms (SNPs) of the FSHR show reduced responsiveness of antral follicles, as assessed by FORT, to FSH administration. FORT was similar for different haplotypes; Thr307-Asn680 (45.9%) and Ala307-Ser680 (39.4%), 307Thr/Ala-Ala/Ala (41.1%; 5.0–91.6%) versus 307Thr/Thr (44.4%; 17.3–83.3%); and in 680Asn/Ser-Ser/Ser (40.0%; 5.0–91.6%) versus 680Asn/Asn (42.2%; 8.3–90.0%) carriers. The authors concluded that antral follicle responsiveness to FSH, as far as measured by FORT, is not influenced by the presence of SNPs of FSHR 307Ala and 680Ser [73]. The results of another prospective study investigating the association of FSHR polymorphisms with ovarian response in a large population of Chinese women receiving ART (*N* = 450) concluded that subjects with AA or SS genotypes have higher basal FSH levels and have an increased risk of poor response compared with carriers of other genotypes (*p* < 0.05) [74]. Finally, in their systematic review and meta-analysis, Alviggi et al. sought to define the impact of seven polymorphisms of the FSHR, LHR and LHCGR on ovarian stimulation outcomes. More oocytes were retrieved from FSHR (rs6165) AA homozygotes than from GG homozygotes and AG heterozygotes, and stimulation duration was shorter in AA homozygotes than in AG carriers. Furthermore, a higher number of oocytes and MII oocytes were observed in AA than in GG homozygote carriers, with FSH consumption significantly lower in FSHR (rs1394205) GG homozygotes and AG heterozygotes than in AA homozygotes [46].

**Statement 16: Androgen supplementation before ovarian stimulation could be considered in selected POSEIDON patients.**

This statement received 61% agreement from the extended panel, which was below the threshold for consensus. The reasons for disagreement are listed in Supplementary Table 2.

**Statement 16 (Re-vote): The existing evidence is not enough to support the use of androgen supplementation or other adjuvant therapy in POSEIDON patients.**

Following discussion regarding the lack of reliable evidence from RCTs and prospective studies to support the use of androgen supplementation or other adjuvant therapy in POSEIDON patients, the wording was revised and received 84% agreement after re-voting.

Several randomized controlled trials that assessed the effect of dehydroepiandrosterone (DHEA) supplementation on IVF outcomes have reported different outcomes for pregnancy and live birth. The first RCT, which compared the effect of DHEA supplementation on IVF treatment outcomes among a cohort of women with known decreased ovarian reserve, showed that long-term androgen priming with DHEA improved IVF outcomes in patients with a prior poor response to COS. Specifically, a significantly higher number of oocytes were retrieved from women who received DHEA supplementation (25 mg three times daily [t.i.d]. for ≥ 12 weeks before starting COS) (*n* = 67) compared with those who did not (*n* = 66) (*p* < 0.001). There was also a significantly lower cancellation rate (*p* < 0.01), a higher number of embryos transferred (*p* < 0.001) and a higher pregnancy rate per cycle (*p* < 0.05) in patients who received DHEA compared with the control group. However, although numerally higher in the study group, there was no statistically significant difference in pregnancy rate (per ET) between the two groups [75]. In a later RCT (a double-blind, randomized, placebo-controlled study) evaluating the effect of pretreatment with DHEA on IVF outcomes in women of AMA (36–40 years) and normal ovarian reserve, the women who received 75 mg of DHEA once a day 8 weeks prior to ovulation induction and up to their β-HCG test (*n* = 53) had a significantly higher LBR compared with those who received placebo during this period (*n* = 56) (*p* < 0.05) [76]. Finally, a systematic review and meta-analysis of RCTs that compared ovarian response and/or pregnancy outcomes between the different IVF protocols using androgens (DHEA and testosterone) and conventional IVF stimulation in patients with diminished ovarian reserve and/or poor ovarian responders, reported no statistical difference when DHEA priming was compared with placebo or no pretreatment [77]. Testosterone pretreatment yielded a higher number of oocytes retrieved (mean difference, 0.94; 95% CI 0.46–1.42), a higher clinical pregnancy rate (risk ratio, 2.07; 95% CI 1.33–3.20), and higher LBR (risk ratio, 2.09; 95% CI 1.11–3.95). However, the results of this analysis should be interpreted with caution, owing to the low-to-moderate quality of the available evidence, the high level of heterogeneity in the studies reporting on testosterone treatment (only one study reported a significant effect on embryo number) and the merged classification criteria of women with diminished ovarian reserve and/or poor ovarian responders defined according to the Bologna criteria, rather than poor responders classified according to the POSEIDON criteria.

A recent retrospective, cohort study investigated the potential effects of DHEA supplementation on the IVF outcomes of patients who fulfilled the criteria for POSEIDON Group 4. The intervention group comprised 159 cycles, with patients receiving DHEA daily (30 mg t.i.d.) for 12 weeks before their IVF cycles. The control group included 138 cycles with patients who underwent IVF cycles but did not receive DHEA. Patients who received DHEA had a significantly higher FOI and higher numbers of retrieved oocytes, MII oocytes, fertilized oocytes, Day 3 embryos and top-quality Day 3 embryos than those in the control group (all *p* ≤ 0.001). Higher cumulative pregnancy rates (29.5% vs. 14.8%; *p* = 0.011) and lower cancellation rates (18.9% vs. 29.7%; *p* = 0.029) were observed in the DHEA group versus the control group; however, clinical pregnancy rate, LBR and CLBR did not differ between the two groups. Regardless of age (≤ 40 or > 40 years), higher numbers of oocytes and embryos were observed in the intervention versus the control group; however, in patients aged > 40 years, the cumulative pregnancy rate was significantly higher in those who received DHEA versus those who did not (31.2% vs. 10.6%; *p* = 0.022) [78]. The lack of consistent evidence was acknowledged by Orvieto (2022) in his review of the literature on the use of pretreatment therapies (including androgen supplementation) in patients with prior poor ovarian response. He concluded that further, large, prospective studies are required to validate the specific mode/combination of pretreatment measures and identify the specific characteristics of women who might benefit from pretreatment prior to ovarian stimulation [79]. Adding to the disparity among studies, a cross-sectional study of 1423 women aged 18–75 years randomly recruited from the community setting over 15 months documented the effects of age and natural and surgical menopause on androgen levels in healthy women. In the reference population (*n* = 595) total testosterone, free testosterone, DHEA sulfate and androstenedione levels declined steeply with age (*p* < 0.001), with the decline of each being greater in the earlier than the later decades; in contrast, lower total and free testosterone levels were observed in women aged ≥ 55 years who reported bilateral oophorectomy [80].

Importantly, after the Delphi consensus process was completed, a multicenter, multinational Phase 3 double-blind placebo-controlled RCT of 290 women with POR aged 18–43 years was published (the T-Transport Study). The authors found that testosterone pretreatment (5.5 mg) did not increase the number of oocytes retrieved (3.24 ± 2.25 vs. 3.69 ± 2.72), number of MII oocytes (2.75 ± 2.06 vs. 2.83 ± 1.91), number of embryos (1.46 ± 1.22 vs. 1.90 ± 1.92) or clinical pregnancy rate (17.42% vs. 16.30%) compared with placebo, respectively [81].

Among the other adjuvant treatments proposed so far for women meeting the POSEIDON criteria, the most promising findings were reported in a retrospective analysis that investigated the effect of growth hormone (GH) in women with poor ovarian reserve (AMH < 1.2 ng/mL) undergoing IVF/ICSI treatment. Women were allocated to either GH-adjuvant or non-adjuvant (GH + and GH-, 338 cycles in each) and further split into POSEIDON Groups 3 (age < 35 years) and 4 (age ≥ 35 years). Overall, adjuvant GH showed a beneficial effect on the ovarian response and LBR in patients with poor ovarian reserve. Further stratification revealed that in POSEIDON Group 4, there was a significantly increased number of good-quality embryos in the GH group compared with those not receiving GH group (1.58 ± 1.71 vs. 1.25 ± 1.55, *p* = 0.032), accompanied by a reduced miscarriage rate and an increase in LBR (29.89 vs. 17.65%, *p* = 0.028); however, adjuvant GH failed to improve the LBR in POSEIDON Group 3 patients [82].

Building on the data from this study, in a retrospective study of 428 low-prognosis women with a previous failed IVF/ICSI cycle who received GH in the subsequent cycle commenced within 12 months and stratified according to the POSEIDON criteria based on age and AMH, Liu et al. (2021) reported improvements in outcomes across all POSEIDON groups for those receiving adjuvant GH. The live birth rates were 47.66%, 28.33%, 45.45%, and 24.07% and the ORs of clinical pregnancy were 19.16 (95% CI 7.87–46.63, *p* < 0.001), 7.44 (95% CI 1.65–33.55, *p* = 0.009), 10.19 (95% CI 2.39– 43.52, *p* = 0.002) and 27.63 (95% CI 4.46–171.11, *p* < 0.001) in Groups 1, 2, 3, and 4, respectively, compared with the non-GH cycle. The number of oocytes retrieved was significantly elevated in the subgroups with normal ovarian reserve (incidence rate ratios [IRR] 1.47 [95% CI 1.36–1.59], *p* < 0.001] for Group 1 and IRR 1.31 (95% CI 1.15–1.49, *p* < 0.001] in Group 2, and the number of Day-3 good-quality embryos was significantly elevated in the subgroups with either normal ovarian reserve or younger age (IRR 2.13 [95% CI 1.78– 2.56, *p* < 0.001] for Group 1, IRR 1.54 [95% CI 1.26–1.89, *p* < 0.001] for Group 2, and IRR 1.47 [95% CI 1.10–1.98, *p* = 0.010] for Group 3 [83].

A summary of the role of interventions in patients classified according to the POSEIDON criteria has been reported by Esteves et al. [84] (Supplementary Tables 3 & 4). Nonetheless, despite this evidence, there are currently not enough sufficiently robust data to support the routine use of any adjuvant strategy in women meeting the POSEIDON criteria.

**Statement 17: Oocyte or embryo accumulation are valid strategies in POSEIDON patients with low ovarian reserve**.

This statement achieved 72% agreement from the extended panel. Accumulation of oocytes from several ovarian stimulation cycles using vitrification technologies is a novel strategy for managing patients with a poor response to ovarian stimulation that has been reported in several cohort and observational studies [85, 86]. A prospective cohort study assessed the efficiency of a new strategy that takes advantage of vitrification as a means to create larger cohorts of oocytes. The cohort comprised 242 low-responder (LR) patients (594 cycles) whose mature oocytes were accumulated by vitrification and inseminated simultaneously (LR-Accu-Vit) and 482 patients (588 cycles) undergoing IVF-ET with fresh oocytes in each stimulation cycle (LR-fresh). The ET cancellation per patient was significantly lower in the LR-Accu-Vit group compared with the LR-fresh group (*p* < 0.05). Furthermore, LBR was numerically higher (but not statistically significant) in the LR-Accu-Vit group versus the LR-fresh group, but CLBR was significantly higher in the LR-Accu-Vit group than in the LR-fresh group (*p* < 0.05) with a similar, but not statistically significant, outcome observed among patients aged ≥ 40 years [87]. A single-arm pilot study investigated the efficacy of double ovarian stimulation during both the follicular and luteal phases in patients with poor ovarian response undergoing IVF and ICSI. Thirty-eight women meeting the ESHRE Bologna criteria initiated mild ovarian stimulation. After the first oocyte retrieval, hMG and letrozole were administrated to stimulate follicle development, and oocyte retrieval was carried out a second time when the dominant follicles had matured. For the primary outcome measure of oocytes retrieved, a total of 167 oocytes were collected and 26 out of 38 women (68.4%) produced one to six viable embryos, which were cryopreserved for later transfer. Of these, 21 women underwent 23 FET, resulting in 13 clinical pregnancies [88]. A prospective paired non-inferiority observational study compared the euploid blastocyst formation rates per injected MII oocyte following follicular phase versus luteal phase stimulation performed in the same menstrual cycle (DuoStim) in 51 women with reduced ovarian reserve undergoing preimplantation genetic diagnosis for aneuploidy testing (PGD-A). There were no statistically significant differences between the number of retrieved cumulus-oocyte complexes (5.1 ± 3.4 vs. 5.7 ± 3.3), MII oocytes (3.4 ± 1.9 vs. 4.1 ± 2.5) or biopsied blastocysts per stimulated cycle (1.2 ± 1.2 vs. 1.4 ± 1.7) from follicular versus luteal stimulation, respectively. Furthermore, euploid blastocyst rates per biopsied blastocyst or per injected MII oocyte were similar among follicular and luteal stimulations [89].

Other studies have investigated the potential of DuoStim in women with low ovarian reserve. In an observational study of poor responder patients fulfilling the ESHRE Bologna criteria, 100 out of 297 patients chose to undergo DuoStim to measure the contribution of luteal phase stimulation to the CLBR per intention-to-treat (ITT). Among the 100 patients who completed DuoStim, the CLBR per ITT increased from 7% after follicular phase stimulation to 15% after DuoStim. Conversely, the CLBR per ITT among the 197 patients who chose a conventional COS strategy was 8%, since only 17 patients who were not pregnant returned for a second stimulation after the first attempt (drop-out rate, 91%) [90]. A systematic review by Vaiarelli et al. summarized the evidence to date for the use of the DuoStim protocol to improve fertility treatment outcomes. Although the quality of the 21 clinical studies included in the qualitative synthesis was moderate–low, all of them highlighted that dual stimulation in the same ovarian cycle is a valid option to increase the number of oocytes retrieved. The authors concluded that DuoStim is a promising unconventional stimulation protocol but it must be standardized, with more robust studies performed to determine the true clinical advantages and disadvantages of its implementation in IVF [91]. This latter point was reiterated by Tocci et al., who reported that the use of DuoStim currently lacks rationale, evidence, and follow-up, and unless valid clinical indications have been established, DuoStim should be only used in controlled clinical trials with appropriate experimental consents [92].

## Discussion

This Delphi consensus provides real-world clinical perspectives from a diverse international group of experts, who have compiled a series of literature-supported consensus statements regarding the overall relevance of the POSEIDON criteria and clinical implications on the diagnosis of infertility in women with POR. These statements covered a wide range of focus areas, comprising overall relevance, impact of female age, biomarkers relevance and thresholds, oocyte number, prevalence, low prognosis validation, primary outcome in POSEIDON studies, ART Calculator importance, role of the FOI index, management of POSEIDON Group 2 and management of POSEIDON patients.

The relevance of the POSEIDON criteria was addressed in Statement 1, in which the experts acknowledged that the criteria are timely and clinically sound when considered in the context of ‘the ability to retrieve the number of oocytes necessary to obtain at least on euploid embryo’. Accordingly, the criteria can improve counseling and management of low prognosis patients undergoing ART, with an expected positive effect on reproductive success and a reduction in the time to live birth. Critical data are now needed from prospective trials to confirm that patient-oriented strategies to achieve the POSEIDON measure of success increase the continuum of reproductive outcomes, including the time to live birth. Randomized trials to clarify the role of interventions in this vast and important group of ART patients are currently ongoing [9].

Statement 2 addressed the impact of female age, with consensus reached on 35 years as an acceptable threshold to distinguish between young and old low-prognosis patients. This was based on the wealth of published evidence that supports this threshold, while acknowledging that an additional important threshold could be defined when women reach > 40 years [18]. The critical data on the validity of applying this age threshold in the POSEIDON criteria are currently being collected in clinical trials.

The panel agreed that AMH and AFC have a fundamental role in identifying patients with poor prognosis, and the thresholds stipulated in the POSEIDON criteria are probably sufficient. They also recommended that the biomarker that best meets the physician’s clinical setting should be used, owing to the low predictive value of POSEIDON for low oocyte yield (Statements 3 and 4). The panel also acknowledged the high degree of variability in AFC measurement among operators and in AMH measurement depending on the assay used; therefore, the criteria may need periodic adjustment as assay and ultrasound instrumentation improve. Furthermore, the addition of new biomarkers into updates on the criteria could improve the prediction of which women will be categorized into Group 1 and 2.

The panel unanimously agreed that there is particularly strong evidence that number of oocytes retrieved is a prognostic factor for live birth in both fresh cycles and for CLBR (Statement 5). CLBR is acknowledged as a complete measure of success of an IVF treatments, and improvements in oocyte culture to the blastocyst stage and cryopreservation (i.e., by vitrification) have been implicated in the significant increase in CLBR per oocyte aspirated (from 27.0 to 36.3%) between 2007 and 2017 [93]. However, there is still inconsistency in identifying the most appropriate parameters required to calculate cumulative live birth rate [39]. In response to this, the POSEIDON investigators have proposed guidance on uniform terminology and metrics for use in research studies, including study design, information to include when reporting studies using the POSEIDON criteria, endpoints and the use of the ART Calculator [94].

A considerable proportion of patients (40–60%) treated in fertility clinics would meet the POSEIDON criteria (Statement 6), although this range may vary and more data are needed, particularly from Europe and the USA, to gain insight into the true number. The panel were in agreement that the true value of the POSEIDON criteria for low prognosis validation (low CLBR per aspirated IVF/ICSI cycle compared with normal responders) lies in the combined assessment of oocyte quality and quantity, rather than quantity alone (Statement 7). Furthermore, the panel strongly agreed that CLBR was the best primary outcome among POSEIDON patients or between POSEIDON and non-POSEIDON patients, owing to the increase in freeze-all cycles and frozen ET (Statement 8).

The panel considered the benefits of two new tools that can assist in determining the number of oocytes required to achieve live birth: the ART Calculator and the FOI. The panel acknowledged that by determining the number of oocytes needed as a function of age (and number of euploid embryos), the ART Calculator is of great help in counselling couples and determining the number of oocytes that will be needed (Statement 9). However, as the calculator was validated using retrospective data from ART centers, the predictive value remains to be tested in large prospective studies, particularly with respect to live birth outcomes. Similarly, the panel agreed that the FOI is an excellent marker for assessing the dynamics of follicle growth as an index of ovarian sensitivity to gonadotropins, with the aim to improve ovarian stimulation in subsequent cycles (Statement 10). However, as is the case for the ART Calculator, FOI still needs to be tested in large prospective trials in different patient subsets.

Of the POSEIDON subgroups, there is a wealth of evidence showing the benefit of r-hLH supplementation from Day 1 in older patients, owing to the age-related effect on the number and sensitivity of gonadotropin receptors in granulosa cells (Statement 11) [95]. This may be particularly relevant for women in POSEIDON Group 2 (i.e., older women with normal ovarian reserve). However, a distinction needs to be made between those patients who receive r-hLH and those who are treated with LH-like activity derived from human chorionic gonadotropin (hCG), as the two molecules activate different signaling pathways following binding to LHCGR [96, 97].

Several statements address the overall management of POSEIDON patients (Statements 12–17) and highlight potential strategies to be tested in RCTs. The application of pharmacogenomics to ovarian stimulation is one way to optimize ovarian response to support oocyte or embryo accumulation and decrease patient drop out. Recombinant gonadotropins may be considered for POSEIDON patients, owing to the higher number of oocytes retrieved compared with urinary preparations, and higher doses of the recombinant product may be considered in POSEIDON Groups 1 and 2 (depending on the initial dose), to counteract any potential reduced affinity for the FSHR, specifically in women with the Ser/Ser680 polymorphism [98]. In addition, supplementation with r-hLH may also improve results in normogonadotropic women with hyporesponse, as well as specifically for women in Group 2. Currently, there is not enough evidence to support the use of androgen supplementation or other adjuvant therapies.

### Strengths

The participants were fertility experts from across the globe, representing different regions, including Europe, Asia, North America and South America, reflecting the quality of healthcare and different approaches to infertility treatment in different parts of the world. Additionally, there was a high level of agreement from the extended panel, with three statements achieving 100% agreement, eight statements achieving > 90% and three statements achieving > 80% agreement after the first round of voting. Notably, only one statement (Statement 16) failed to reach consensus during the first round of voting by the extended panel, although this statement achieved 84% consensus after rewording and revoting. Lastly, a number of peer-reviewed studies have been identified to support each of the statements.

### Limitations

In addition to the strengths of this consensus, we also acknowledge that this study is limited as the statements presented only represent the collective opinion of the experts included and are not exhaustive.

## Conclusions

This Delphi consensus provides a far-reaching overview of the overall relevance of the POSEIDON criteria to identify potential clinical implications and measure its impact on the diagnosis of infertility in women with POR, which can now be verified in prospective studies as the next step to realize the full potential of the POSEIDON criteria.

## Electronic supplementary material

Below is the link to the electronic supplementary material.

Supplementary Material 1

## Data Availability

Any requests for data by qualified scientific and medical researchers for legitimate research purposes will be subject to Merck KGaA’s Data Sharing Policy. All requests should be submitted in writing to Merck KGaA’s data sharing portal https://www.merckgroup.com/en/research/our-approach-to-research-and-development/healthcare/clinical-trials/commitment-responsible-data-sharing.html. When Merck KGaA has a co-research, co-development, or co-marketing or co-promotion agreement, or when the product has been out-licensed, the responsibility for disclosure might be dependent on the agreement between parties. Under these circumstances, Merck KGaA will endeavor to gain agreement to share data in response to requests.

## Abbreviations

AFC: Antral follicle count
AMA: Advanced maternal age
AMH: Anti-müllerian hormone
ART: Assisted reproductive technology
CGH: Comparative genomic hybridization
CI: Confidence interval
CLBR: Cumulative live birth rate
COS: Controlled ovarian stimulation
DHEA: Dehydroepiandrosterone
ESHRE: European Society of Reproduction and Embryology
ET: Embryo transfer
FOI: Follicle-to-Oocyte Index
FORT: Follicle output rate
FSH: Follicle-stimulating hormone
FSHR: Follicle-stimulating hormone receptor
GH: Growth hormone
GnRH: Gonadotropin hormone-releasing hormone
HP-hMG: Highly purified human menopausal gonadotropin
ICSI: Intracytoplasmic sperm injection
IVF: In-vitro fertilization
LBR: Live birth rate
LHB: Luteinizing hormone beta subunit
LHCGR: Luteinizing hormone chorionic gonadotropin receptor
LR: Low responder
MII: Metaphase II
NGS: Next-generation sequencing
PGT-A: Preimplantation genetic testing for aneuploidy
POR: Poor ovarian responder
POSEIDON: Patient-oriented strategies encompassing individualized oocyte number
POR: Poor ovarian responder
RCT: Randomized controlled trial
r-hFSH: Recombinant human follicle-stimulating hormone
r-hLH: Recombinant human luteinizing hormone
ROC: Receiver operating characteristic
SNP: Single nucleotide polymorphism
t.i.d: Three times daily
