# Supplementary material for: Patients with low prognosis in ART: a Delphi consensus to identify potential clinical implications and measure the impact of POSEIDON criteria
Source: Reprod Biol Endocrinol. 2024 Oct 10;22:122. doi: 10.1186/s12958-024-01291-x (PMC11465546; doi:10.1186/s12958-024-01291-x)
Supplement: Supplementary file 1 — Supplementary Material 1 [file 12958_2024_1291_MOESM1_ESM.docx]

## Supplementary Table 1: Participants included in the extended panel

| **Name** | **Affiliation** |
| --- | --- |
| Baris Ata | Department of Obstetrics, Gynecology and Reproductive Sciences, Yale School of Medicine, New Haven, Connecticut, USA |
| Ariel Weissman | Department of Obstetrics and Gynecology, Wolfson Medical Center, Holon, Israel; Affiliated to the Sackler Faculty of Medicine, Tel Aviv University, Tel Aviv, Israel |
| Carolo Dosuoto | Department of Obstetrics, Gynecology and Reproductive Medicine, Santa Creu I Sant Pau Hospital, Puigvert Foundation, Barcelona, Spain |
| Antoine Abu-Musa | American University of Beirut Medical Center, Beirut, Lebanon |
| Thor Haahr | Department of Clinical Medicine – Department of Obstetrics and Gynecology, Denmark |
| Budi Wiweko | Division of Reproductive Endocrinology and Infertility, Department of Obstetrics and Gynecology, Faculty of Medicine, Universitas Indonesia Jakarta, Indonesia; Yasmin IVF Clinic, Dr. Cipto Mangunkusumo General Hospital, Jakarta, Indonesia; Human Reproductive, Infertility and Family Planning Research Cluster, Indonesia Medical Education and Research institute (IMERI), Faculty of Medicine, Universitas Indonesia, Jakarta, indonesia |
| Sonia Malik | Southend Fertility and IVF Center, New Delhi, India; Max Smart Super Speciality Hospital, Saket, New Dehli, India |
| Michael Grynberg | Department of Reproductive Medicine & Fertility Preservation, Antoine Béclère University Hospital, Clamart, France |
| Joaquin Liàcer | Ginefiv, Madrid, Spain |
| Josè Teixeira da Silva | Centre for Reproductive Genetics Alberto Barros (CGR), Av. Do Bessa, 240, 1° Dto. Frente, 4100-009 Porto, Portugal |
| John Yovich | Department of Medical Sciences, Curtin University, Perth, Australia; Cairns Fertility Centre, Queensland, Australia |
| Claus Y Andersen | Department of Clinical Medicine, Faculty of Health and Medical Sciences, University of Copenhagen, Denmark |
| Panagiotis Drakopoulos | Centre for Reproductive Medicine, UZ Brussel, Belgium |
| Shu Foong | Regional Fertility Program, NW, Calgary, Canada |
| Evangelos Papanikolaou | Assisting Nature IVF Centre and Genetics, Thessaloniki, Greece |
| Margarida Silvestre | Coimbra Fertility Center, Coimbra, Portugal |
| Nguyen Khanh Linh | IVFMD, My Duc Phu Nhuan Hospital, Ho Chi Minh City, Vietnam |
| Le Long Ho | IVFMD, My Duc Phu Nhuan Hospital, Ho Chi Minh City, Vietnam |
| Ha Nhat Anh | IVFMD, My Duc Hospital, Ho Chi Minh City, Vietnam |
| Bülent Urman | Department of Women Health and In Vitro Fertilization, American Hospital, Istanbul, Turkey; Department of Gynecology and Obstetrics, Hacettepe University, Ankara, Turkey |
| Sezcan Mumusoglu | Department of Obstetrics and Gynecology, School of Medicine, Hacettepe University, Ankara, Turkey |
| Roy Homburg | Hewitt Centre for Reproductive Medicine, Liverpool Women's NHS Foundation Trust, Liverpool, UK |
| Neri Laufer | Department of Obstetrics and Gynecology, Hadassah Medical Center, Jerusalem, Israel |
| Pasquale Patrizio | Department of Obstetrics, Gynecology and Reproductive Sciences, University of Miami, Miller School of Medicine, Miami, Florida, USA |
| Klaus Bühler | Scientific Clinical Centre for Endometriosis, University Hospitals of Saarland, Saarbrüken, Germany; Department of Gynaecology, Jena-University Hospital-Friedrich Schiller University, Jena, Germany |
| Jean Noel Hughes | Department of Obstetrics and Gynaecology, Center for Reproductive Medicine, Jean Verdier Hospital, University Paris XIII, Paris, France |
| PC Wong | Division of Reproductive Endocrinology and Infertility, Department of Obstetrics and Gynecology, National University Hospital, Singapore |
| Shahar Kol | IVF Unit, Elisha Hospital, Haifa Area, Netanya, Center District, Israel |
| Neena Malhotra | All India Institute of Medical Sciences, Delhi, India |
| Pedro Xavier | Centre for Reproductive Genetics, Porto, Portugal; Department of Gynecology and Obstetrics, São João Hospital, University of Porto, Porto, Portugal |
| Fernando Neuspilier | IVI Buenos Aires, Buenos Aires, Argentina |
| Giuliano Bedoschi | Department of Obstetrics and Gynecology, Ribeirao Preto School of Medicine, University of Sao Paulo, Ribeirao Preto, Brazil |
| Thi Minh Chau Le | Department of Infertility, Tu Du Hospital, Ho Chi Minh City, Vietnam |
| Juan Antonio Garcia Velasco | Department of Reproductive Endocrinology and Infertility, IVI Madrid, Rey Juan Carlos University, Madrid, Spain |
| Andrea Borini | Technobios Procreazione, Centre for Reproductve Health, Bologna, Italy |
| Christophe Blockeel | Centre for Reproductive Medicine, UZ Brussel, Belgium |
| Alberto Vaiarelli | IVIRMA, Global Research Alliance, GENERA, Clinica Valle Giulia, Roma, Italy |
| Amergio Vitagliano | Department of Interdisciplinary Medicine (DIM), Unit of Obstetrics and Gynecology, University of Bari “Aldo Moro”, policlinico of Bari, Bari, Italy |
| Dolors Manau | Assisted Reproduction Unit, Hospital Clinic de Barcelona, Barcelona, Spain |
| Juan Josè Espinòs | Reproductive Endocrinology Unit, Hospital de la Santa Creu i Sant Pau, Barcelona, Spain |

## Supplementary Table 2: Statements with more than 20% disagreement during Round 2.

|  | Disagreements |
| --- | --- |
| Statement 12: The use of recombinant gonadotropins may be considered for women fulfilling the POSEIDON criteria because they result in an increased oocyte number | - r-hFSH appears to be associated with an increase in the number of oocytes obtained, although this effect on the cumulative delivery rate is not as obvious when compared with urinary formulation. - The results of different trial show variable results. |
| Statement 16: Androgen supplementation before ovarian stimulation could be considered in selected Poseidon patients | - We do not have for the moment strong evidence from RCTs and we do not know the exact dose and duration of administration. - As Orvieto states, large prospective studies that should validate the specific mode/combination of pretreatment measures are needed. - Although it can be considered, my opinion is that evidence for its widespread use in the cases described is still lacking. - We have to wait for new studies and the question is: which androgen - The result of ongoing RCTs should be waited to make such a conclusion. - It depends on the source of androgens, i.e. recent RCT showing that DHEA is of no benefit https://obgyn.onlinelibrary.wiley.com/doi/10.1111/1471-0528.17045. - No evidence |

**Supplementary table 3: Summary evidence of trials examining the role of interventions in patients classified as POSEIDON groups 1 or 2**

| **Reference** | **Study type** | **Country** | **OS characteristics** | **Population** | **Intervention** | **Comparison** | **Outcome** | **Results** |
| --- | --- | --- | --- | --- | --- | --- | --- | --- |
| Drakopoulos  et al., 2018 | Observational  case-control | Belgium, Spain,  Portugal,  Croatia, and  Denmark  (multicenter) | Fixed GnRH antagonist protocol  Conventional ovarian  stimulation with rec-FSH 150-225 IU/day  hCG trigger  Inclusion criteria: women <40  years | N = 160  patients  Groups 1b and  2b (number per group not  specified) | Same or  increased FSH  dose on 2nd IVF cycle | First IVF cycle  outcomes on  same patients | Stimulation  Duration  No. oocytes  No. embryos | 1st cycle: 9.6 ± 2.4  2nd cycle: 9.5 ± 1.8  (p = NS)  1st cycle: 6.5 ± 1.6  2nd cycle: 9.3 ± 4.8  (p < 0.01)  1st cycle: 2.9 ± 1.6  2nd cycle: 4.0 ± 3.0  (p < 0.01) |
| Eftekar et al.,  2020 | Observational  case-control | Iran (single center) | GnRH antagonist protocol  rec-FSH 150 IU/day + GnRH-a trigger (FFOS)  hMG (300 IU/day) + hCG trigger (LPOS) | N = 10 Group 1a | Follicular phase ovarian stimulation  (FFOS) | Luteal phase  ovarian  stimulation  (LPOS) in the  same cycle and same patient  subjected to  FFOS | No. oocytes  Retrieved  No. MII oocytes  No. embryos  MII rate  Fertilization  rate | FFOS: 1.9 ± 1.1  LLOS: 9.2 ± 6.8  (p = 0.004)  FFOS: 1.7 ± 0.8  LLOS: 7.9 ± 5.0  (p = 0.016)  FFOS: 1.3 ± 0.5  LLOS: 4.8 ± 2.8  (p = 0.013)  FFOS: 94.2%  LLOS: 78.1%  (p = 0.038)  FFOS: 90.5%  LLOS: 53.7%  (p = 0.009) |
| Li et al., 2020 | Observational  cohort | China (single center) | Conventional ovarian stimulation with rec-FSH alone in doses ranging from 112.5 to  300 IU/day  hCG trigger | N = 3342 IVF ICSI cycles  Group 1: n = 1326;  Group 2: n = 767;  Group 3: n = 410*;  Group 4: n = 839* | Early-follicular phase long acting  GnRH long protocol (EFLL) | Mid-luteal phase  Short acting  GnRH-a long protocol (MLSL)  GnRH antagonist  Protocol (GnRH- ant) | Stimulation  duration (days)  No. oocytes  retrieved  Implantation  Live birth rate  per transfer | Group 1:  EFLL: 13.5 ± 2.1  MLSL: 11.3 ± 1.9  GnRH-ant: 11.2 ± 2.7  (p < 0.05: EFLL vs. MLSL and GnRH-ant groups)  Group 2:  EFLL: 13.4 ± 2.1  MLSL: 11.1 ± 1.9  GnRH-ant: 10.3 ± 2.4  (p < 0.05: EFLL vs. MLSL and GnRH-ant groups)  Group 1:  EFLL: 12.8 ± 6.4  MLSL: 10.8 ± 5.8  GnRH-ant: 8.7 ± 7.1  (p < 0.05: EFLL vs. MLSL and GnRH-ant groups; p < 0.05: MLSL vs. GnRH-ant)  Group 2:  EFLL: 7.7 ± 3.5  MLSL: 8.1 ± 5.0  GnRH-ant: 4.9 ± 3.2  (p < 0.05: EFLL and  MLSA vs. GnRH-ant)  Group 1:  EFLL: 42.8%  MLSL: 40.3%  GnRH-ant: 37.9%  (p = NS)  Group 2:  EFLL: 19.8%  MLSL: 17.1%  GnRH-ant: 28.7%  (p = NS)  Group 1:  EFLL: 39.7%  MLSL: 30.7%  GnRH-ant: 26.5%  (p < 0.05: EFLL vs. MLSL and GnRH-ant groups)  Group 2:  EFLL: 13.4%  MLSL: 12.4%  GnRH-ant: 12.4% (NS) |
| Farimani et al.,  2021 | Observational  case-control | Iran (single center) | Shanghai protocol | N = 96 patients  Group 1: n = 7;  Group 2: n = 17;  Group 3: n = 16*  Group 4: n = 56* | Intraovarian  platelet-rich  plasma (PRP) | Ovarian  stimulation  parameters of  follicular phase  stimulation in  same patients  on the same  cycle | No. oocytes  retrieved  (median)  No, MII oocytes | Group 1:  Pre-PRP: 4 ± 2; post-  PRP: 9 ± 5 (p < 0.05)  Group 2: Pre-PRP: 2 ± 3; post- PRP: 5 ± 5 (p < 0.05)  Group 1:  Pre-PRP: 2 ± 3; post-  PRP: 8 ± 3 (p < 0.05)  Group 2: Pre-PRP: 2 ± 3; post-PRP: 4 ± 3.5 (p < 0.05) |
| Cozzolino et al., 2021 | Observational  cohort | Spain (multicenter) | Conventional ovarian stimulation (cOS) consisting of  a flexible GnRH antagonist and  200-300 IU/day rec-  FSH + 75IU HP-HMG  Minimal ovarian stimulation (mOS) consisted of oral daily clomiphene citrate (50-100 mg) for 4 days, followed  by HP-HMG or FSH (150 IU) every other day + flexible GnRH  antagonist OCP used before stimulation in  both protocols  Trigger carried out with rec-hCG | N = 2002 patients  Group 2: n = 1519;  Group 4: n = 483* | mOS | cOS | Stimulation  Days  No. oocytes  retrieved  No. MII oocytes  No. embryos  Cumulative  delivery rate  per started  cycle | mOS: 11.2 ± 1.8  cOS: 10.5 ± 1.6  (p < 0.001)  mOS: 7.9 ± 4.6  cOS: 11.9 ± 6.5  (p < 0.001)  mOS: 6.1 ± 4.1  cOS: 9.1 ± 5.3  (p < 0.001)  mOS: 4.6 ± 3.1  cOS: 6.6 ± 4.3  (p < 0.001)  mOS: 31.1% (52/167)  cOS: 36.9% (524/1417)  (OR: 1.29, 95% CI 0.91-1.87; p = 0.15) |
| Du et al., 2021 | Observational  cohort | China (single center) | Conventional ovarian stimulation with hMG or u-FSH  at a dose of 150-300 IU/ day + hCG trigger | N = 1329 patients  Group 1: n = 250;  Group 2: n = 511;  Group 3: n = 111*;  Group 4: n = 457* | Progestin primed ovarian stimulation  (PPOS) | Flexible GnRH  antagonist (F- GnRHa) | No. oocytes  Cumulative live birth rate per aspiration cycle | Group 1:  PPOS: 4.7 ± 2.7;  F-GnRHa: 4.0 ± 2.5  (p = NS)  Group 2:  PPOS: 4.2 ± 2.4;  F-GnRHa: 3.7 ± 2.4  (p = NS)  Group 1:  PPOS: 54.4%;  F-GnRHa: 53.8%  (p = NS)  Group 2:  PPOS: 18.1%;  F-GnRHa: 24.3%  (p = NS) |
| Zhang et al.,  2021 | Observational;  retrospective | China (single center) | Conventional ovarian stimulation with hMG or u-FSH at a dose of 150-300 IU/day + hCG trigger | N = 920 cycles  (835 patients)  Group 1: n = 148;  Group 2: n = 276;  Group 3: n = 183*;  Group 4: n = 313*  Inclusion criteria:  Age 40;  BMI ≤25 | Progestin primed ovarian stimulation (PPOS) | Flexible GnRH  antagonist  (F-GnRHa) | Cumulative live  birth rate per  aspiration cycle | Group 1:  PPOS: 23.7%;  F-GnRHa: 31.9%  (p = NS)  Group 2:  PPOS: 22.5%;  F-GnRHa: 32.0%  (p = NS) |
| Abbreviations: Rec-hCG: recombinant human chorionic gonadotropin; rec-FSH: recombinant follicle-stimulating hormone; u-FSH: urinary follicle-stimulating hormone; HP-hMG: highly purified human menopausal gonadotropin; hMG: human menopausal gonadotropin; GnRH: gonadotropin releasing-hormone; OCP: oral contraceptive pill; IU: international units; IVF: in vitro fertilization; ICSI: intracytoplasmic sperm injection; NS: not significant; OR: odds ratio.  Table adapted from: S.C. Esteves, H. Yarali, L.N. Vuong et al., POSEIDON groups and their distinct reproductive outcomes: Effectiveness and cost-effectiveness insights from real-world data research, Best Practice & Research Clinical Obstetrics and Gynaecology, https://doi.org/10.1016/j.bpobgyn.2022.05.003 | | | | | | | | |

**Supplementary table 4: Summary evidence of trials examining the role of interventions in patients classified as POSEIDON groups 3 or 4**

| **Reference** | **Study type** | **Country** | **OS characteristics** | **Population** | **Intervention** | **Comparison** | **Outcome** | **Results** |
| --- | --- | --- | --- | --- | --- | --- | --- | --- |
| Huang et al.,  2018 | Observational  cohort | China (single center) | GnRH agonist and  GnRH antagonist  protocols  Conventional ovarian stimulation with hMG rec-hCG trigger | N = 1233 patients  Group 3: n = 337;  Group 4: n = 896 | GnRH agonist protocol (GnRH-a) | GnRH antagonist protocol (GnRH-ant) | No. oocytes  Implantation  Rates  Live birth rate  per transfer | Group 3  GnRH-a: 4.4 ± 2.5  GnRH-ant: 4.9 ± 2.5 (p = NS)  Group 4 (36-39 years)  GnRH-a: 4.0 ± 2.3  GnRH-ant: 4.3 ± 2.2 (p = NS)  Group 4 ( 40 years)  GnRH-a: 3.2 ± 1.7  GnRH-ant: 3.5 ± 2.1 (p = NS)  Group 3:  GnRH-a: 25-3%  GnRH-ant: 10.7%  (p = 0.03)  Group 4 (36-39 years)  GnRH-a: 19.4%  GnRH-ant: 16.9%  (p = NS)  Group 4 ( 40 years)  GnRH-a: 6.6%  GnRH-ant: 5.0%  (p = NS)  Group 3:  GnRH-a: 30.8%  GnRH-ant: 16.7%  (p = 0.06)  Group 4 (36-39 years)  GnRH-a: 24.9%  GnRH-ant: 26.7%  (p = NS)  Group 4 ( 40 years)  GnRH-a: 4.5%  GnRH-ant: 4.0%  (p = NS) |
| Xu et al., 2018 | Randomized open-label controlled trial | China (single center) | Flexible GnRH  antagonist protocol  Conventional ovarian stimulation with rec-FSH (225 IU/d) + hMG (225 IU/day)  rec-hCG trigger | N = 186  patients (Group 3) | Oral coenzyme  Q10 (CoQ10)  200 mg three  timed a day for  60 days before  ovarian  stimulation | Cycles in which  CoQ10 was not  used (no  placebo used) | Gonadotropin dose; total (IU); median  No of oocytes retrieved; median  No of high quality embryos obtained; median†  % of cancelled cycles (lack of embryos for transfer)  Cumulative live birth rate  Stimulation duration (days)  No. oocytes retrieved  Miscarriage rate  Live birth delivery rate per transfer | CoQ10: 2000 (IQR: 1200-4275)  Control: 3075 (IQR: 1900-4275); p = 0.03  CoQ10: 4 (IQR: 2-5)  Control: 2 (IQR: 1-4); p = 0.002  CoQ10: 1 (IQR: 0-2)  Control: 0 (IQR: 0-1.75); p = 0.002  CoQ10: 8.3% (14/76)  Control: 22.9%;  p = 0.04  CoQ10: 28.9%  Control: 15.5%;  p = 0.08  Group 3: GH: 9.7 ± 2.3 Control: 10.8 ± 1.9 (p < 0.001)  Group 4: GH: 9.9 ± 2.0 Control: 10.7 ± 2.0 (p < 0.001)  Group 3: GH: 3.9 ± 2.8 Control: 4.4 ± 3.0 (p = NS)  Group 4: GH: 3.5 ± 2.8 Control: 3.1 ± 2.6 (p = NS)  Group 3: GH: 0.0%  Control: 26.9%  (p = NS)  Group 4: GH: 18.2%  Control: 60.0%  (p = 0.005)  Group 3: GH: 29.6%  Control: 28.8%  (p = NS)  Group 4: GH: 27.3%  Control: 9.2%  (p = 0.003) |
| Chen et al. 2019 | Observational  case-control | Taiwan (single center) | Flexible GnRH  antagonist protocol  Conventional ovarian stimulation with rec-FSH alone, rec-FSH + rec-LH, or hMG, in doses ranging from  112.5 to 300 IU/day  Rec-hhCG trigger or  dual trigger (rec-hCG + leuprolide) | N = 297 cycles  (Group 4) | DHEA (30 mg three times per day) daily for 12 weeks before IVF | Cycles in which DHEA was not used | Stimulation duration  No. oocytes  Retrieved  No. MII oocytes  Live birth rate  per cycle  Cumulative live birth rate per  aspiration cycle | DHEA: 10.7 ± 2.3  Control: 10.3 ± 2.4 (p = NS)  DHEA: 3.1 ± 2.8  Control: 1.9 ± 1.8  (p < 0.001)  DHEA: 2.6 ± 2.1  Control: 1.7 ± 1.5  (p < 0.001)  DHEA: 11.9%  Control: 9.4%  (p = NS)  DHEA: 20.0%  Control:12.0%  (p = NS) |
| Li et al., 2020 | Observational  cohort | China (single center) | Conventional ovarian stimulation with rec-FSH alone in doses ranging from 112.5 to 300 IU/day hCG trigger | N = 3342 cycles  Group 1: n = 1326  Group 2: n = 767  Group 3: N = 410  Group 4: N = 839 | Early-follicular phase long acting  GnRH-long protocol  (EFLL) | Mid-luteal phase  Short acting GnRH-a  long protocol (MLSL)  GnRH antagonist  Protocol (GnRH- ant) | Stimulation  duration (days)  No. oocytes  Retrieved  Implantation  Live birth rate per transfer | Group 3:  EFLL: 14.0 ± 2.5  MLSL: 11.9 ± 3.2  GnRH-ant: 10.2 ± 2.8 (p < 0.05: EFLL vs. MLSL and GnRH-ant groups; p < 0.05: MLSL vs. GnRH-ant)  Group 4: EFLL: 14.6 ± 2.5 MLSL: 12.5 ±  3.0 GnRH-ant: 9.4 ± 2.4 (p <0.05: EFLL vs. MLSL and GnRH ant groups; p < 0.05: MLSL vs. GnRH-ant)  Group 3: EFLL: 5.6 ± 3.6 MLSL: 4.6 ± 3.2  GnRH-ant: 2.4 ± 1.7 (p < 0.05: EFLL vs.MLSL and GnRH-ant groups; p < 0.05: MLSL vs. GnRH-ant)  Group 4: EFLL: 3.9 ± 2.8 MLSL: 3.1 ± 2.3  GnRH-ant: 2.5 ± 1.8 (p < 0.05:  EFLL and MLSA vs. GnRH-ant; ant)  Group 3: EFLL: 38.8% MLSL: 32.8%  GnRH-ant: 37.3% (p = NS)  Group 4: EFLL: 18.1% MLSL: 16.4%  GnRH-ant: 20.3%  (p = NS)  Group 3:  EFLL: 31.4% MLSL: 23.4% GnRH-ant: 12.6% (p < 0.05: EFLL vs. MLSL and GnRH-ant groups;  p < 0.05: MLSL vs. GnRH-ant)  Group 4: EFLL: 9.7%  MLSL: 7.3% GnRH-ant: 7.0% (p = NS) |
| Chern et al., 2020 | Observational  cohort | Taiwan (single center) | Flexible GnRH  antagonist protocol  Conventional ovarian stimulation with either rec-FSH alone or combined with rec-LH or hMG | N = 252 frozen thawed embryo transfer cycles  performed in  patients of  group 4 | Dual trigger (rec-hCG + GnRH agonist) | Rec-hCG trigger | No. oocytes  Retrieved  Implantation  Rate  Live birth rate/transfer | Dual trigger: 3.3 ± 2.7 hCG trigger: 1.6 ± 1.5 (p < 0.001)  Dual trigger: 14.4% ± 30.0 hCG trigger: 5.4% ± 18.8  (p = 0.004)  Dual trigger: 17.5%  hCG trigger: 5.4%  (p = 0.006)  Dual-trigger was positively  associated with live birth rate after adjustments for  confounders (OR = 3.16, 95% CI  1.06-9.38, p = 0.039) |
| Tandulwadkar  & Karthick 2020 | Pilot study |  | Mini-long GnRH  agonist protocol and  hMG (300-450 IU/day) rec-hGG trigger | N = 20 patients  of groups 3 & 4  No. patients per  group not specified | Laparoscopic/  transvaginal  intraovarian  instillation of  autologous  bone marrow derived stem  cells combined  with plateletrich  plasma | Ovarian  markers  recorded 6  months before  the  intervention | AFC  No, oocytes retrieved | Pre-treatment: 3.35 ± 0.98  After treatment: 5.7 ± 1.75 (p = 0.0001)  Pre-treatment: not reported After treatment: 4.0 ± 1.6 |
| Berker et al., 2021 | Observational  cohort | Turkey (single  center) | Flexible GnRH  antagonist protocol  Conventional ovarian stimulation with rec-FSH, in doses ranging  from 225 to 450 IU/day rec-hCG trigger | N = 558 patients  (groups 3 and 4)  No. patients per  group not specified | hMG added (75-150 IU/d) from early  follicular phase or midfollicular phase | Rec-FSH | Stimulation  duration (days)  No. oocytes  Retrieved  Live birth rate per embryo transfer | Early hMG: 11.9 ± 3.6  Mid-hMG: 12.8 ± 4.0  Rec-FSH: 12.5 ± 5.1 (p = 0.03:  early-hMG vs. mid-hMG and  rec-FSH groups)  Early hMG: 1.6 ± 1.7  Mid-hMG: 1.6 ± 1.7  Rec-FSH: 1.9 ± 1.8  (p = NS)  Early hMG: 21.9%  Mid-hMG: 11.7%  Rec-FSH: 11.6% (p = 0.03: early hMG  vs. mid-hMG and rec-FSH groups) |
| Liu et al., 2021 | Observational  cohort | China (single center) | GnRH agonist and  GnRH antagonist  protocols  Conventional ovarian stimulation with rec-FSH or a combination of rec-FSH and hMG  hCG trigger | N = 380 patients  Group 3: n = 137  Group 4: n = 243 | GnRH agonist | GnRH  antagonist | Cumulative live  birth delivery  per started  cycle | Group 3: GnRHa: 52.7% GnRH-ant: 36.9% (p = 0.06)  Group 4: GnRHa: 26.9% GnRH-ant: 13.7% (p = 0.01)  In group 4, patients with higher AMH levels ( 0.785 ng/ml)  who received the GnRH agonist long protocol achieved  significantly higher cumulative live birth rate than who  received the GnRH antagonist protocol |
| Farimani et al., 2021 | Observational  case-control | Iran (single center) | Shanghai protocol | N = 96 patients  Group 1: n = 7  Group 2: n = 17  Group 3: n = 16  Group 4: n = 56 | Intraovarian  platelet-rich  plasma (PRP) | Ovarian stimulation  parameters of follicular phase stimulation in same patients on the same cycle | No. oocytes  retrieved  (median)  No. MII oocytes  (median) | Group 3: Pre-PRP: 1 ± 3; post-PRP: 2 ± 7 (p < 0.05)  Group 4: Pre-PRP: 1 ± 1; post-PRP: 3 ± 4 (p < 0.001)  Group 3: Pre-PRP: 1 ± 3; post-PRP: 2 ± 6.5 (p < 0.05)  Group 4: Pre-PRP: 1 ± 2; post-PRP: 2 ± 3.7 (p < 0.001) |
| Orvieto et al., 2021 | Proof of concept | Israel (single center) | GnRH agonist from  midluteal phase until menses  Letrozole (5 days after stopping the GnRH agonist)  High dose  gonadotropin  stimulation with FSH (LH added on  stimulation day 6  onwards)  GnRH antagonist (from stimulation day 6 until day before trigger)  Dual trigger (hCG +  GnRH-agonist) | N = 5 patients  (group 4)  Inclusion criteria: previous failed IVF/ICSI cycles  with conventional ovarian stimulation | Modified stop GnRH-agonist  (GnRH-ag) protocol  combined with aromatase  inhibitor priming | Previous IVFICSI  cycle (within a 3-  month period)  of same patient | No. oocytes  retrieved | Stop GnRH-ag + letrozole priming: 3.8 ± 2.4;  Conventional stimulation: 2.0 ±  1.2 (p = 0.04) |
| Cozzolino et al., 2021 | Observational  cohort | Spain (multicenter) | Conventional ovarian stimulation (cOS) consisting of a flexible GnRH antagonist protocol and 200-300  IU/day rec-FSH + 75IU HP-HMG  Minimal ovarian  stimulation (mOS)  consisted of oral daily clomiphene citrate (50-100 mg) for 4 days, followed by HP-HMG or FSH (150 IU) every other day + flexible GnRH antagonist  OCP used before  stimulation in both  protocols  Trigger carried out with  rec-hCG | N = 2002 patients  Group 2: n = 1519  Group 4: n = 483 | mOS | cOS | Stimulation days  No. oocytes retrieved  No. MII oocytes  No. embryos  Cumulative  delivery rate per started cycle | mOS: 11.1 ± 2.3  cOS: 11.0 ± 2.4  (p = NS)  mOS: 2.9 ± 1.9  cOS: 3.7 ± 2.4  (p < 0.001)  mOS: 2.1 ± 1.1  cOS: 2.8 ± 2.1  (p < 0.001)  mOS: 0.8 ± 1.6  cOS: 0.9 ± 1.2  (p = NS)  mOS: 11.4% (28/245)  cOS: 11.1% (34/308)  (OR 0.96, 95% CI 0.55-1.70; p = NS) |
| Du et al., 2021 | Observational  cohort | China (single center) | Conventional ovarian stimulation with hMG or u-FSH at a dose of 150-300 IU/day hCG trigger | N = 1329 patients  Group 1: n = 250  Group 2: n = 511  Group 3: n = 111  Group 4: n = 457 | Progestin primed ovarian stimulation  (PPOS) | Flexible GnRH  antagonist (F-GnRHa) | No. oocytes  Cumulative live birth rate per  aspiration cycle | Group 3: PPOS: 2.8 ± 2.0; F-GnRHa: 2.8 ± 2.1 (p = NS)  Group 4: PPOS: 1.6 ± 1.4; F-GnRHa: 1.6 ± 1.3 (p = NS)  Group 3: PPOS: 16.9%; F-GnRHa: 23.5% (p = NS)  Group 4: PPOS: 10.5%; F-GnRHa: 7.4% (p = NS) |
| Zhang et al., 2021 | Observational;  retrospective | China (single center) | Conventional ovarian stimulation with hMG or u-FSH at a dose of 150-300 IU/day  hCG trigger | N = 920 cycles  (835 patients)  Group 1: n = 148  Group 2: n = 276  Group 3: n = 183  Group 4: n = 313  Inclusion criteria:  Age 40; BMI ≤25 | Progestin primed  ovarian stimulation  (PPOS) | Flexible GnRH antagonist (F-GnRHa) | Cumulative live birth rate per  aspiration cycle | Group 3: PPOS: 24.0%; F-GnRHa: 29.1% (p = NS)  Group 4: PPOS: 29.0%; F-GnRHa:  42.9% (p = 0.02) |
| Song et al., 2021 | Randomized  controlled trial | China (multicenter) | Flexible GnRH  antagonist protocol  Conventional ovarian stimulation with rec-FSH 150-300 IU/d + rec-LH  Dual trigger (0.2 mg  triptorelin + 250 mcg rec-hCG) | N = 462 patients (group 4) | Traditional  Chinese formula Ding-Kun pill (DPK)  given twice daily from day 5 of previous menstrual cycle until oocyte  retrieval | Placebo | No. oocytes  Retrieved (median [IQR])  No. embryos available for transfer (median [IQR])  Ongoing pregnancy rate (OPR) per  randomized patient | DPK: 6.0 [3.0]; Placebo: 6.5 [4.0]  (p = NS)  DPK: 3.0 [3.0]; Placebo: 4.0 [4.0]  (p = NS)  DPK: 26.4%; Placebo: 24.2%  (p = NS) |
| Abbreviations: Rec-hCG: recombinant human chorionic gonadotropin; rec-FSH: recombinant follicle-stimulating hormone; FSH: follicle-stimulating hormone; LH: luteinizing hormone;  HP-hMG: highly purified human menopausal gonadotropin; hMG: human menopausal gonadotropin; GnRH: gonadotropin releasing-hormone; OCP: oral contraceptive pill; DHEA: Dehydroepiandrosterone;  IVF: in vitro fertilization; ICSI: intracytoplasmic sperm injection; IU: international units; NS: not significant; IQR: interquartile range; OR: odds ratio.  Note: High quality embryos defined as embryos that reached 6 to 8-cell stage with cytoplasmic fragmentation of less than 10% of the embryo surface and equal size blastomeres.  Table adapted from: S.C. Esteves, H. Yarali, L.N. Vuong et al., POSEIDON groups and their distinct reproductive outcomes: Effectiveness and cost-effectiveness insights from real-world data research, Best Practice & Research Clinical Obstetrics and Gynaecology, https://doi.org/10.1016/j.bpobgyn.2022.05.003 | | | | | | | | |
